# A G316A Polymorphism in the Ornithine Decarboxylase Gene Promoter Modulates MYCN-Driven Childhood Neuroblastoma

**DOI:** 10.3390/cancers13081807

**Published:** 2021-04-09

**Authors:** Laura D. Gamble, Stefania Purgato, Michelle J. Henderson, Simone Di Giacomo, Amanda J. Russell, Paolo Pigini, Jayne Murray, Emanuele Valli, Giorgio Milazzo, Federico M. Giorgi, Mark Cowley, Lesley J. Ashton, Jaydutt Bhalshankar, Gudrun Schleiermacher, Ali Rihani, Tom Van Maerken, Jo Vandesompele, Frank Speleman, Rogier Versteeg, Jan Koster, Angelika Eggert, Rosa Noguera, Raymond L. Stallings, Gian Paolo Tonini, Kwun Fong, Zalman Vaksman, Sharon J. Diskin, John M. Maris, Wendy B. London, Glenn M. Marshall, David S. Ziegler, Michael D. Hogarty, Giovanni Perini, Murray D. Norris, Michelle Haber

**Affiliations:** 1 Children’s Cancer Institute, Lowy Cancer Research Centre, UNSW Australia, PO Box 81, Randwick, NSW 2031, Australia; lgamble@ccia.org.au (L.D.G.); mhenderson@ccia.org.au (M.J.H.); jmurray@ccia.org.au (J.M.); evalli@ccia.org.au (E.V.); mcowley@ccia.org.au (M.C.); Glenn.Marshall@health.nsw.gov.au (G.M.M.); dziegler@ccia.org.au (D.S.Z.); MNorris@ccia.unsw.edu.au (M.D.N.); 2 Department of Pharmacy and Biotechnology, University of Bologna, 40126 Bologna, Italy; stefania.purgato2@unibo.it (S.P.); simone.digiacomo2@unibo.it (S.D.G.); paolo.pigini2@unibo.it (P.P.); giorgio.milazzo2@unibo.it (G.M.); federico.giorgi@unibo.it (F.M.G.); giovanni.perini@unibo.it (G.P.); 3 Cancer Research Program, The Garvan Institute of Medical Research, Darlinghurst, NSW 2010, Australia; a.russell@garvan.org.au; 4 Research Portfolio, University of Sydney, Sydney, NSW 2008, Australia; lesley.ashton@sydney.edu.au; 5 SIREDO, Department of Paediatric, Adolescents and Young Adults Oncology and INSERM U830, Institut Curie, 26 rue d’Ulm, 75005 Paris, France; jaydutt.bhalshankar@curie.fr (J.B.); gudrun.schleiermacher@curie.fr (G.S.); 6 Center for Medical Genetics, Ghent University, C. Heymanslaan 10, 9000 Ghent, Belgium; ali.rihani@ki.se (A.R.); Tom.VanMaerken@UGent.be (T.V.M.); jo.vandesompele@ugent.be (J.V.); franki.speleman@ugent.be (F.S.); 7 Department of Oncogenomics, Academic Medical Center, University of Amsterdam, 1100 Amsterdam, The Netherlands; r.versteeg@amc.uva.nl (R.V.); jankoster@amsterdamumc.nl (J.K.); 8 Department of Pediatric Hematology, Oncology and SCT, Charité-University Hospital Berlin, Campus Virchow-Klinikum, 10117 Berlin, Germany; angelika.eggert@charite.de; 9 Department of Pathology, Medical School, University of Valencia, 46010 Valencia, Spain; Rosa.Noguera@uv.es; 10 CIBERONC-INCLIVA, Biomedical Health Research Institute, 46010 Valencia, Spain; 11 Molecular and Cellular Therapeutics, Royal College of Surgeons in Ireland, D02 YN77 Dublin 2, Ireland; rstallings@rcsi.ie; 12 Neuroblastoma Laboratory, Fondazione Istituto di Ricerca Pediatrica Città della Speranza, 35127 Padova, Italy; gp.tonini@irpcds.org; 13 Thoracic Research Centre, University of Queensland, The Prince Charles Hospital, Brisbane, QLD 4032, Australia; fongk@health.qld.gov.au; 14 Division of Oncology and Center for Childhood Cancer Research, Children’s Hospital of Philadelphia, Philadelphia, PA 19104, USA; vaksmanz@email.chop.edu (Z.V.); diskin@email.chop.edu (S.J.D.); Maris@email.chop.edu (J.M.M.); hogartym@email.chop.edu (M.D.H.); 15 Department of Pediatrics, Perelman School of Medicine, University of Pennsylvania, Philadelphia, PA 19104, USA; 16 Dana-Farber/Boston Children’s Cancer and Blood Disorders Center, Harvard Medical School, Boston, MA 02215, USA; wendy.london@childrens.harvard.edu; 17 Kids Cancer Centre, Sydney Children’s Hospital, High St, Randwick, NSW 2031, Australia; 18 Centre for Childhood Cancer Research, University of New South Wales, Sydney, NSW 2052, Australia

**Keywords:** ODC1, MYCN, SNP, neuroblastoma

## Abstract

**Simple Summary:**

Neuroblastoma is a devasting childhood cancer in which multiple copies (amplification) of the cancer-causing gene MYCN strongly predict poor outcome. Neuroblastomas are reliant on high levels of cellular components called polyamines for their growth and malignant behavior, and the gene regulating polyamine synthesis is called ODC1. ODC1 is often coamplified with MYCN, and in fact is regulated by MYCN, and like MYCN is prognostic of poor outcome. Here we studied a naturally occurring genetic variant or polymorphism that occurs in the *ODC1* gene, and used gene editing to demonstrate the functional importance of this variant in terms of ODC1 levels and growth of neuroblastoma cells. We showed that this variant impacts the ability of MYCN to regulate ODC1, and that it also influences outcome in neuroblastoma, with the rarer variant associated with a better survival. This study addresses the important topic of genetic polymorphisms in cancer.

**Abstract:**

Ornithine decarboxylase (ODC1), a critical regulatory enzyme in polyamine biosynthesis, is a direct transcriptional target of MYCN, amplification of which is a powerful marker of aggressive neuroblastoma. A single nucleotide polymorphism (SNP), G316A, within the first intron of *ODC1*, results in genotypes wildtype GG, and variants AG/AA. CRISPR-cas9 technology was used to investigate the effects of AG clones from wildtype *MYCN*-amplified SK-N-BE(2)-C cells and the effect of the SNP on MYCN binding, and promoter activity was investigated using EMSA and luciferase assays. AG clones exhibited decreased *ODC1* expression, growth rates, and histone acetylation and increased sensitivity to ODC1 inhibition. MYCN was a stronger transcriptional regulator of the *ODC1* promoter containing the G allele, and preferentially bound the G allele over the A. Two neuroblastoma cohorts were used to investigate the clinical impact of the SNP. In the study cohort, the minor AA genotype was associated with improved survival, while poor prognosis was associated with the GG genotype and AG/GG genotypes in *MYCN*-amplified and non-amplified patients, respectively. These effects were lost in the GWAS cohort. We have demonstrated that the *ODC1* G316A polymorphism has functional significance in neuroblastoma and is subject to allele-specific regulation by the MYCN oncoprotein.

## 1. Introduction

Neuroblastoma is a childhood cancer that often presents as high-risk disease that is resistant to treatment. Amplification of the *MYCN* oncogene occurs in 20–25% of cases, and is a powerful and reliable marker of poor prognosis [1]. MYCN belongs to the MYC family of transcription factors and functions by forming heterodimers with MAX that typically bind six nucleotide ‘E-box’ sequences at the promoters of target genes.

Polyamines are organic cations that play critical roles in a number of cellular processes and abnormalities in the control of polyamine metabolism and transport can result in increased polyamine levels promoting tissue proliferation and tumor formation [2]. Ornithine decarboxylase (ODC1) is a critical regulatory enzyme in the polyamine biosynthesis pathway and is a direct transcriptional target of MYC oncoproteins [3,4]. It is the first enzyme in polyamine synthesis and is the rate limiting step for the conversion of ornithine to the primary polyamine, putrescine. ODC1 is essential for normal development and tissue repair in mammals but is downregulated in most adult tissues [5].

*ODC1* has been implicated as an important gene during the early stages of tumor progression and is highly expressed in a variety of cancer types [6,7,8,9,10]. High *ODC1* expression is found in *MYCN*-amplified neuroblastoma and is associated with a poor prognosis [10,11]. However, high *ODC1* expression also predicts poor outcome in *MYCN* non-amplified neuroblastoma suggesting it has oncogenic abilities independent of MYCN [10,11]. Disabling ODC1 using the specific inhibitor difluoromethylornithine (DFMO) inhibits neuroblastoma proliferation in vitro as well as in transgenic and patient derived xenograft mouse models of neuroblastoma [10,11,12,13] and based on these findings, clinical trials are being conducted combining DFMO with conventional chemotherapy and/or chemoimmunotherapy in relapsed/refractory neuroblastoma patients (ClinicalTrials.gov Identifier: NCT02030964 and NCT03794349).

A single nucleotide polymorphism (SNP, rs2302615) in the regulatory region of *ODC1*, located in the proximal region of intron 1 +316 nucleotides 3′ of the transcriptional start site (chr2(hg19):g.10588138C > T) has been identified (Figure S1) [14]. This SNP, G316A, is located between neighboring E-boxes that cooperate to influence *ODC1* promoter activity [3,14,15], while the sequences flanking these E-boxes have been shown to influence binding of MYC [16,17]. Three possible genotypes result from the SNP: GG, AG and the variant AA which occurs at a low frequency of 7–10% in the general population [14,18]. A previous study has shown that the transcriptional repressor Mad1 suppresses the activity of the *ODC1* promoter in colon cancer cells containing the A-allele but not the G-allele, and other studies have also reported that the E-box repressors MAD4 (MXD4) and MXI1 bind less strongly in cells homozygous for the GG genotype [18,19]. These findings suggest allele-specific regulation of *ODC1* by E-box transcription factors. It has also been shown that the GG genotype is associated with improved survival compared to people with AG/AA genotypes in colorectal and breast cancer patients [18,19]. This finding contrasts with those from a colon cancer prevention trial, in which individuals with the AA genotype who reported using aspirin were 10× less likely to have an adenoma recurrence compared to non-aspirin users with the GG genotype [20,21].

In this study, we investigated both the functional and prognostic significance of the G316A SNP in neuroblastoma cell lines and tumors. We demonstrate that the *ODC1* G316A polymorphism influences ODC1 expression, neuroblastoma cell growth and response to DFMO. In contrast to studies in adult cancer patients the G allele was associated with a poorer outcome in neuroblastoma patients. In addition, MYCN preferentially binds the G allele over the A allele, and produces a greater stimulatory effect on the *ODC1* promoter in the presence of the G allele.

## 2. Results

### 2.1. The 316A Allele Is Associated with Reduced Cell Growth and ODC1 Expression, and Increased Sensitivity to DFMO in Cas9-Edited Neuroblastoma Cells

**Figure 1:**
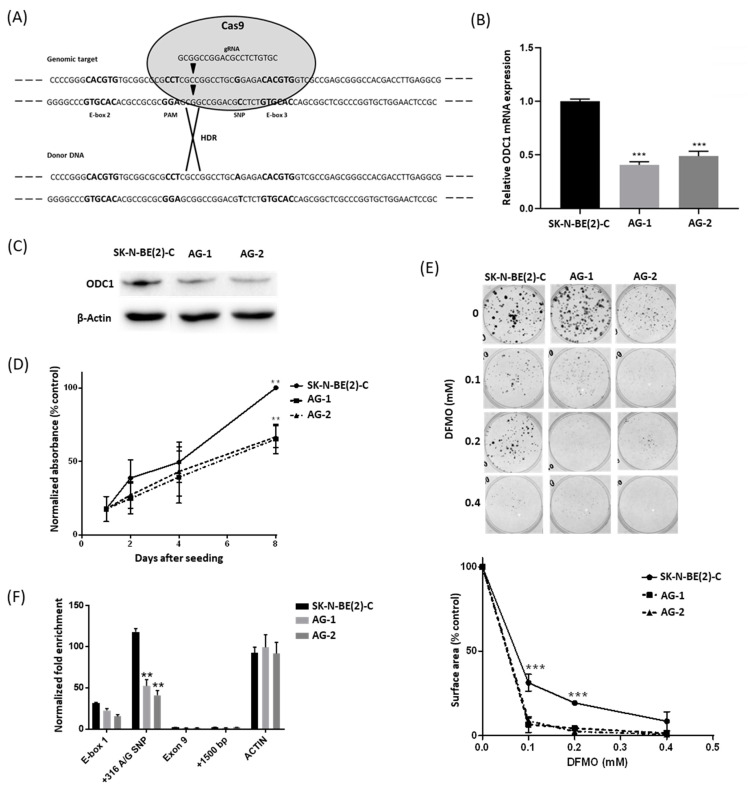
Analysis of AG clones generated from the GG SK-N-BE(2)-C cell line by CRISPR-Cas9 editing. (**A**) The CRISPR-Cas9 editing strategy was adopted to edit SK-N-BE(2)-C cells. The gRNA cleavage site (inverted triangles) is located 9 bp upstream of the G316A SNP. The donor DNA sequence carries the A SNP mutation (black). (**B**,**C**) ODC1 expression analysis by qRT-PCR (**B**) and Western blot (**C**) of CRISPR-edited clones AG-1 and AG-2, compared to parental SK-N-BE(2)-C cells. qRT-PCR data were normalized using GUSB as reference gene, and standardized as previously described [22]. Β-actin was used as the loading control for the Western blot. Uncropped WB images are available in Figure S10. (**D**) Proliferation rates of CRISPR-edited clones. Cell growth was measured by BrdU assay. Absorbance data were normalized to day 1 and SK-N-BE(2)-C was used as the control. (**E**) Analysis of the effect of DFMO on CRISPR-edited clones by clonogenic assay. The total area occupied by cell colonies was measured by crystal violet staining and subsequent ImageJ analysis. All *p* values were determined by one-way ANOVA tests and compared to SK-N-BE(2)-C as control. (**F**) ChIP analysis of H3-histone acetylation of the G316A SNP spanning region in the CRISPR-edited clones. The analyzed sequences in the ODC1 locus are E-box1, G316A SNP, Exon 9 and a sequence located +1500 bp downstream of the locus. Acetylation of a region in the Actin gene was used as a control. Enrichment data were normalized to a non-acetylated region located 15,000 bp upstream of the ODC1 locus. A representation of the analyzed region is shown in Figure S3A. ** *p* < 0.005, *** *p* < 0.001. All experiments were performed at least 3 times.

We utilized CRISPR-Cas9 technology to examine the functional effects of the ODC1 SNP genotype in *MYCN*-amplified SK-N-BE(2)-C cells exhibiting the GG genotype. Adopting the HDR-mediated editing strategy (Figure 1A) and donor DNA from neuroblastoma NBL-S cells, which are of AG genotype, we obtained two independent cell lines carrying the AG genotype (AG-1 and AG-2, Figure S2). Both *ODC1* mRNA and protein expression were markedly decreased in AG-1 and AG-2 lines when compared to wildtype cells (Figure 1B,C), and they also had a significantly reduced proliferation rate following 8 days of growth (Figure 1D). Using colony assays, the AG lines were found to be more sensitive to the ODC1 inhibitor DFMO (Figure 1E). Together these observations suggest that the A allele variant reduces *ODC1* transcription, thereby moderating neuroblastoma cell proliferation and influencing response to DFMO-based therapies. Chromatin immunoprecipitation (ChIP) experiments were performed to determine the acetylation status in the region spanning the SNP (construct shown in Figure S3A). Consistent with the expression results, H3 histone acetylation in this region was decreased in AG clones compared to the parental cells (*p* < 0.005), (Figure 1F), demonstrating an important role of the G316A SNP in influencing the chromatin status of the *ODC1* promoter region. Given the decreased level of histone acetylation in the AG clones, we next explored whether decreased MYCN activity at this locus is also associated with the A allele.

### 2.2. The 316A Allele Influences ODC1 Promoter Activity In Vitro

**Figure 2:**
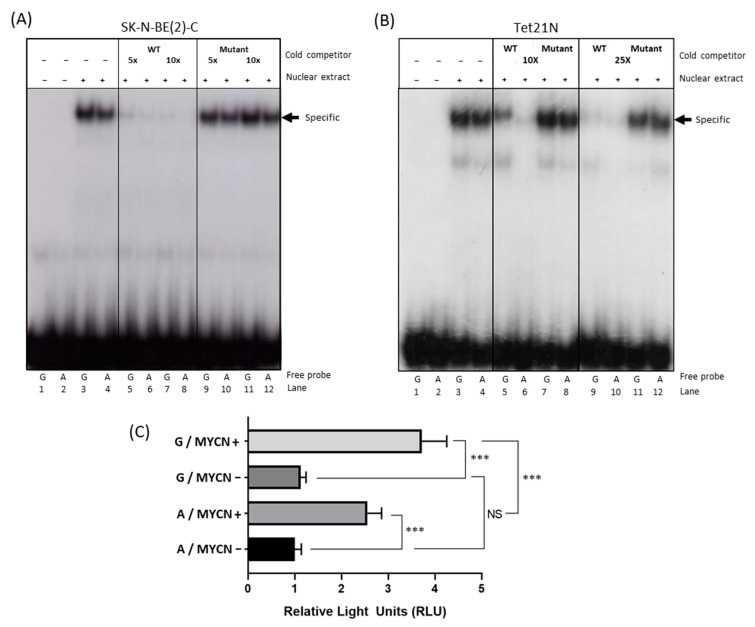
The effect of the ODC1 SNP on MYCN binding and ODC1 promoter activity. (**A,B**) MYCN binding was assessed using EMSA with SK-N-BE(2)-C (**A**) and Tet21N (**B**) nuclear extracts. Nuclear extracts were incubated in the presence of radiolabeled probes containing either the G SNP (G probe) or the A SNP (A probe), and E-box 3. Competition analysis was performed by incubating the binding reaction with 5–10-fold and 10–25-fold molar excess of unlabeled G probe carrying either a wild type (WT) or Mutant E-box. Quantification of the specific bands (arrow) is shown in Figure S4. Note that the order of the lanes differs between A and B. (**C**) Luciferase reporter assays in Tet21N cells transfected with a reporter plasmid containing a region of the ODC1 promoter that includes either the G or the A SNP. Untreated Tet21N cells with MYCN overexpression (MYCN+) were compared to tetracycline-treated cells (MYCN−), where MYCN overexpression is blocked. Replicates were standardized as previously described [22]. A two-way ANOVA was used to test for an interaction between the effects of the SNP and MYCN expression on promoter activity (SNP: F_1,16_ = 20.0, *p* < 0.001; MYCN expression: F_1,16_ = 203.4, *p* < 0.001; Interaction: F_1,16_ = 13.0, *p* = 0.002) followed by Tukey’s post-hoc test for multiple pairwise comparisons. A representation of all the constructs utilized for these experiments are shown in Figure S3B. *** *p* < 0.001.

Electrophoretic mobility shift assays (EMSA) were performed to determine the influence of the SNP genotype on the MYCN/MAX binding properties in the E-box sequence nearest to the polymorphism (E-box 3). Nuclear extracts from SK-N-BE(2)-C and Tet21N cells were incubated with radiolabeled DNA probes containing either the A or the G SNP plus the downstream E-box (Figure S3B). A stronger band was observed for the G probe versus the A probe in the presence of nuclear extracts for both cell lines (Figure 2A,B, lanes 3 and 4), indicating weaker binding of MYCN/MAX to the A probe. This provides further evidence that the A SNP negatively influences *ODC1* expression through reduced binding of the MYCN transcription factor and is consistent with previous data of ours showing that enrichment within the G316A SNP region following MYCN ChIP is approximately two-fold higher in SK-N-BE(2)-C cells, which we have determined to be of GG genotype, than in LAN1 cells, which are of AG genotype [13].

To specifically demonstrate that the bands for the A or the G SNP probes were associated with the MYCN/MAX complex, a competition assay using increasing amounts of cold wild-type or mutated probes for MYCN/MAX was performed. A reduction in binding of the MYCN/MAX complex to the A probe (shown by quantification of band intensity in Figure S4) was more pronounced than that observed with the G probe, demonstrating that MYCN/MAX has higher affinity for the G SNP probe compared to the A probe. This is clearly demonstrated with the lower concentration of the cold wild-type probe in lanes 5 and 6 (Figure 2 and Figure S4). As a negative control, a competition assay was performed using the same probe but containing a mutated canonical E-box and no reductions in band intensity were observed.

The minimal +257/+330 DNA region of the *ODC1* promoter was cloned upstream of a luciferase reporter cassette (Figure S3B) and the resultant vector was transfected into MYCN inducible Tet21N cells. We assessed the influence of the SNP on MYCN-mediated transactivation of *ODC1*. There was a statistically significant interaction between the effects of the SNP and MYCN expression on promoter activity (F(1,16) = 13.0, *p* = 0.002). Tukey’s post hoc tests showed that the promoter activity was strongly enhanced in MYCN-induced cells compared to non-induced cells regardless of genotype. However, consistent with previous findings, *ODC1* promoter activity was significantly reduced in constructs containing the A allele compared to those containing the G allele, following MYCN induction (*p* < 0.001; Figure 2C). Comparing the stimulatory effect of MYCN on *ODC1* promoters containing either allele, there was a 2.9 +/− 0.2-fold increase in luciferase activity for the A allele, and a 3.9 +/− 0.2-fold increase for the G allele suggesting that MYCN stimulates significantly increased levels of *ODC1* promoter activity in the presence of the G allele compared to the A allele (*p* = 0.013).

### 2.3. The ODC1 316A Allele and Outcome in Neuroblastoma Patients

Our study cohort consisted of 839 primary neuroblastoma patient samples recruited from Europe, the USA and Australia prior to 2012, with the majority being recruited much earlier than this. The GWAS cohort from the USA consisted of neuroblastoma patient samples recruited up to 2016. In the study cohort, the AA genotype was found in 61 patients (7.3%), the AG genotype in 272 patients (32.4%), and the GG genotype in 506 patients (60.3%), consistent with population distributions of the three genotypes previously reported (Table S1) [14,18]. The GWAS cohort consisted of 425 patients (8.7%) with AA genotypes, 1963 patients (40.1%) with AG genotypes and 2504 patients (51.2%) with GG genotypes (Table S1). There was an association between tumour stage and genotype (*p* = 0.049), but no association between genotype and age or *MYCN* status in the study cohort. A weak association (*p* = 0.033) between *MYCN* amplification status and genotype was observed in the GWAS cohort (Table 1). As expected for a representative neuroblastoma cohort, stage/risk group, age and *MYCN* status were each prognostic of outcome in both cohorts (*p* < 0.001, Figure S5).

**Figure 3:**
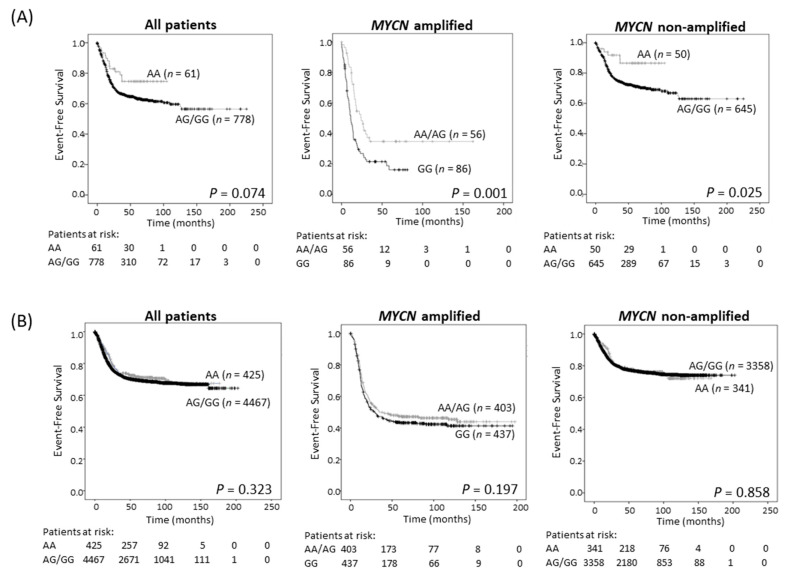
Event-free survival for the 839 neuroblastoma sample study cohort (**A**), and the 4892 patient GWAS cohort (**B**), grouped by genotype (AA vs. AA/AG for all patients and *non-MYCN* amplified patients, and AA/AG vs. GG for *MYCN* amplified patients).

Survival analysis showed improved EFS in the study cohort of neuroblastoma patients with tumors of AA genotype, although this trend did not achieve statistical significance (Table 2; Figure 3A and Figure S6A). Since *ODC1* is an established MYC target gene [3,4], we investigated the possibility of differential effects of the SNP in subsets of patients depending on the *MYCN* status of their tumor. For patients with *MYCN* amplification, the presence of at least one A allele predicted a better outcome than GG homozygous patients (*p* = 0.001, 5-year EFS of 41.1% for AA/AG compared to 22.4% for GG (Figure 3A, Table 2); *p* = 0.003, 5-year OS of 48.2% for AA/AG compared to 28.2% for GG (Figure S6A, Table 2)) and this remained significant following adjustment for age and stage (EFS: *p* = 0.003, OS: *p* = 0.006; Table S2). In patients without *MYCN* amplification, the AA genotype conferred a significantly better EFS, and a trend towards improved OS by comparison with tumors exhibiting an AG/GG genotype (Figure 3A and Figure S6A). These trends were verified in the three separate cohorts that make up the study cohort (Figure S7). Thus, in our study cohort, the impact of the G316A SNP appears to be influenced by MYCN and the adverse effect on outcome conferred by very high levels of MYCN oncoprotein in amplified tumors can be ameliorated by the presence of an A allele. However, in the GWAS cohort, which contains more recent patients who have undergone intensified therapy resulting in improved survival rates of high-risk patients compared to the study cohort (Figure S8), no significant associations between genotype and survival were observed regardless of *MYCN* amplification status (Figure 3B and Figure S6B, Table 2).

### 2.4. A Divergent Role for ODC1 in Adult Cancers

**Figure 4:**
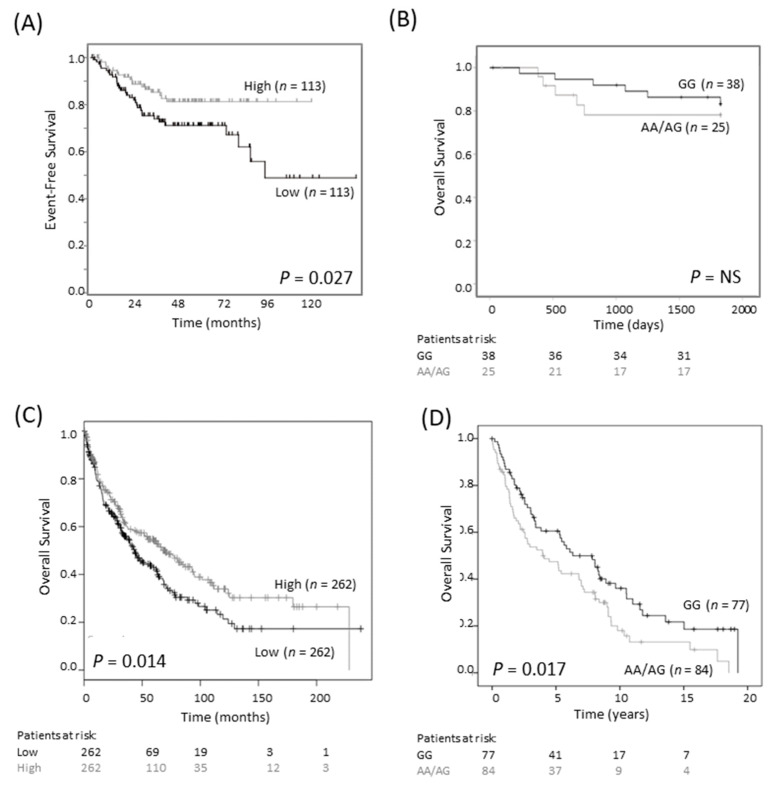
G316A ODC1 promoter SNP and its prognostic significance in colorectal and lung cancer. (**A**) In an online colorectal cancer dataset of 226 primary samples (Sieber dataset), low expression of ODC1 was prognostic of poor outcome (dichotomized at the median). This dataset is available on the R2 platform (R2: Genomics Analysis and Visualization Platform, https://hgserver1.amc.nl/cgi-bin/r2/main.cgi, accessed on 3 August 2020). Patients at risk are not shown as they cannot be generated using this platform. (**B**) In a cohort of 63 rectal cancer patients, where most patients had a favorable outcome, the AA/AG genotype tended to have a poorer overall outcome than the GG genotype. NS—not significant. (**C**) In a lung cancer cohort of 524 squamous cell carcinoma patients, low ODC1 expression is associated with worse outcome. Data was obtained from KM Plotter [23]. (**D**) In a second cohort of 161 squamous cell carcinomas patients, those with AG/AA genotypes tended to have a worse outcome compared with GG genotypes.

Earlier studies on breast and colorectal cancers reported that the G316A GG genotype is associated with a more favorable outcome than the AG/AA genotypes [18,19]. We investigated the association between SNP genotype and survival in a publicly available colorectal cancer cohort of 290 cases (Sieber, R2 Genomics Analysis and Visualization platform; https://hgserver1.amc.nl/cgi-bin/r2/main.cgi, accessed on 3 August 2020). We found that low, rather than high, expression of *ODC1* mRNA was associated with poor EFS (*p* = 0.027, Figure 4A). We also examined a cohort consisting of 63 tumors of rectal origin, since an association between SNP genotype and outcome had previously been reported in this subtype [18]. Despite very few events, there was a trend towards improved survival in patients with the GG genotype compared to patients with AA/AG genotypes (Figure 4B). These findings contrast to the observed association between poor neuroblastoma outcome and high *ODC1* expression, suggesting a different role for ODC1 in colorectal cancer compared with neuroblastoma.

We obtained a similar result in lung cancer patients with small cell carcinoma. Firstly, in a publicly available dataset of 524 patients, low levels of ODC1 mRNA expression were associated with shorter overall survival (Figure 4C). In a cohort of 366 patients with non-small cell lung cancer (NSCLC) of mixed histologies, poorer survival was observed in patients with at least one A allele compared to the GG genotype (*p* = 0.039, Table S3). This relationship was found to be strongest in SCC (*p* = 0.017, Figure 4D) and was maintained following adjustment for ECOG status, advanced tumor stage and age (*p* = 0.049, Table S4). Thus, either low *ODC1* expression or an AG/AA SNP genotype is associated with poor outcome in colorectal and lung cancer. Despite the differing role of ODC1 in neuroblastoma, these findings are consistent with our observations that the A allele is linked to lower *ODC1* expression.

To determine if transcriptional regulators of ODC1 are differentially expressed in the different tissue types, the DoRothEA interactions dataset, available through OmniPath (https://omnipathdb.org/, accessed 27 March 2021 through Bioconductor and RStudio), was explored. The transcription factors (TFs) MYC, MAX, MXD1, MXD4, CREB1 (cAMP-responsive element-binding protein 1), SP1 and WT1 (Wilms’ tumor 1) were identified as regulators of ODC1. Expression of these TFs was explored using the MegaSampler module within the R2 Genomics Analysis and Visualization Platform. As seen in Figure S9A, significant differences in expression across the datasets were observed (one-way ANOVA, *p* < 0.001). Pairwise comparisons showed that, as expected, MYCN was significantly overexpressed in neuroblastoma datasets compared with the adult tumor datasets, whereas the inverse was seen for MYC (Figure S9B). Interestingly, CREB1 was consistently expressed at higher levels in neuroblastoma compared to the other tissues, and WT1 was expressed at lower levels in neuroblastoma compared to most of the adult cancer cohorts. The expression of the other TFs in neuroblastoma compared to the adult cancers was variable. It is therefore possible that the differential SNP effects in the adult cancers, by comparison with neuroblastoma, may be due to differential expression of some of these ODC1-regulating TFs.

## 3. Discussion

Our results show that the A and G alleles of the *ODC1* G316A promoter SNP differentially affect *ODC1* expression, as well as MYCN-mediated *ODC1* transactivation of the E-box region and MYCN oncogenic processes in neuroblastoma cells in vitro. Substituting the A for the G allele in isogenic cells is sufficient to reduce ODC1 mRNA and protein expression and cell proliferation. The underlying molecular mechanism revealed that the A allele has decreased affinity for MYCN, indicating that the region surrounding the E-box is critical in modulating *ODC1* transcriptional function. This is consistent with our previous findings showing a two-fold enrichment in MYCN binding in the region of the ODC1 promoter containing the G316A SNP in SK-N-BE(2)-C cells, which carry the GG genotype, compared with LAN-1 cells, which carry the AG genotype [13]. In addition, compared to cells containing constructs with the A allele, the G allele resulted in greater stimulation of *ODC1* promoter activity by MYCN. These results contrast to previous studies in adult cancers examining this polymorphism [18,19]. In colorectal cancer, studies found that c-MYC had the greatest stimulatory effect on promoters containing the A allele, and interestingly MAD1 was only effective at repressing *ODC1* promoter activity in promoters containing the A allele [18,20]. c-MYC and MXI1 proteins may selectively bind the A-allele in breast cancer, although this study utilized two genetically distinct cell lines rather than isogenic lines [19]. We did not investigate the effect of transcriptional repressors such as MAD1 and MXI1 that, like MYCN, form heterodimers with MAX and bind to E-box sites within the promoters of target genes. Given these previous findings we could speculate that they would also have a greater repressive effect on the *ODC1* promoters containing the G allele. Therefore, the impact of the genotype on *ODC1* expression in neuroblastoma may depend on the balance of MYCN levels and MAD1/MXI1 levels, and their binding with MAX.

Previous studies have found an influence of the G316A SNP on DFMO response. Patients carrying two copies of the G allele had reduced risk of colorectal adenoma recurrence after treatment with DFMO and sulindac, and A allele carriers experienced less treatment-related benefit [24]. We found that Cas9 AG clones are more sensitive to DFMO compared to the parental GG line, which we might expect since the AG clones generate lower levels of ODC1, proliferate at a slower rate and therefore less DFMO is needed to completely block ODC1 protein in these cells. Our findings are in agreement with previous studies in prostate cancer, where, although the ODC1 SNP was not significantly associated with risk, the effect of long-term DFMO treatment on patients with AG/AA genotypes resulted in a reduced prostate volume [25].

Since DFMO is currently in clinical trials for neuroblastoma, our study highlights a subset of patients that may respond differentially to DFMO treatment. The G316A SNP was also studied previously in a DFMO trial involving 18 neuroblastoma patients with relapsed or refractory disease [26]. While there was a tendency towards improved survival for patients with the *ODC1* GG genotype compared to any A allele following DFMO treatment, there was no significant difference, and the effect on urinary polyamines was also not significant. However, the primary aim of the study was to investigate the safety of DFMO in children and given the small number of patients enrolled and the fact that they had received many previous therapies, a large clinical trial is required to address the effect of the G316A SNP in response to DFMO therapy in neuroblastoma.

We demonstrated in our study cohort that the ODC SNP has prognostic significance in *MYCN* amplified and non-amplified neuroblastoma with the AA genotype indicative of a better prognosis. However, this finding did not significantly replicate in the blood-derived DNA in the large 4982 patient GWAS cohort. The rs2303615 genotype in this cohort was not directly measured in the tumor samples by quantitative PCR, but imputed from a genome-wide SNP array in the blood-derived DNA samples and the imputation statistics at rs2302615 were robust. To confirm the discrepancy was not due to the use of an admixed population, we restricted the analysis to Caucasians of European ancestry, but obtained similar results. We also explored the possibility of preferential somatic amplification of the G allele, but saw no evidence for this in the subset of GWAS patients with paired tumor DNA evaluated by SNP arrays or next generation sequencing. It is well known that neuroblastoma patients show significant amounts of tumor-derived DNA in circulation, so we considered the possibility that somatic “contamination” influenced the blood-derived DNA genotyping results. However, we saw no evidence for this after carefully removing blood samples with a clear somatic DNA signature, and also not seeing the same protective effect of the AA genotype in the subjects without *MYCN* amplification. Finally, it is clear that the most important prognostic factor for any cancer patient is response to therapy received. We have shown that the survival rates for high-risk disease are improved in the GWAS cohort compared to the older cohort and thus is likely to have an impact on the influence of the SNP on survival. In addition, *MYCN* amplification status is a robust prognostic marker of outcome, but with the intensification of chemoradiotherapy, recent high-risk neuroblastoma trials showed no impact on outcome for patients whose tumors harbor *MYCN* amplification [27,28], unlike legacy high-risk trials in the past [29]. Since the GWAS cohort was accrued more recently, treatment era might be the best explanation for the discrepant results. Consistent with this, the outcomes in the study cohort are worse than those in the GWAS cohort overall, showing that the outcomes have improved between the timeframes of the two cohorts. Nevertheless, *MYCN* amplification remains an important risk stratification variable and it remains to be seen whether current therapeutic strategies directed towards polyamine depletion will be influenced by this ODC1 polymorphism.

Previous work has demonstrated a protective role for the 316A allele against the recurrence of colon polyps in clinical prevention trials, and the A allele significantly decreased the risk of developing sporadic breast cancer [20,21,30]. However, for breast cancer patients with established disease, the opposite has been found to be true where patients with one or two A alleles had a significantly lower 10-year survival compared to patients with the GG genotype, while colorectal cancer patients with an A allele had a median survival of 81% compared to 89% for GG [18,19]. Neither of these studies addressed whether *ODC1* expression is directly altered by the SNP, although it is known that ODC1 levels are certainly increased compared to normal tissues in both breast and colon cancer [7,31]. The literature surrounding breast and colorectal cancer prognosis in relation to *ODC1* expression is not conclusive [8,32,33,34,35], whereas in neuroblastoma it is well established that high *ODC1* mRNA expression is associated with poor outcome [10,11,13].

Since there are surprisingly few studies looking at *ODC1* expression in adult cancers, we examined a publicly available database and showed that low *ODC1* mRNA expression was prognostic of poor relapse-free survival in colorectal cancer. The finding in our own NSCLC cohort that low mRNA expression of *ODC1* was also associated with worse outcome confirms an earlier report where similar low levels were associated with more aggressive lung tumors [36]. Overall, these data are in contrast with the neuroblastoma results and suggest a unique biology for this MYCN-driven pediatric malignancy where the AA genotype predicts a better outcome and where high ODC1 expression is consistently associated with poor outcome. Interestingly, upon examination of other known ODC1 transcriptional regulators, we found that CREB1 was expressed at higher levels in neuroblastoma compared to other adult tumor types, whereas WT1 was expressed at lower levels. These differences, together with other differences in the pattern of expression of ODC-regulating TFs in neuroblastoma versus other adult cancers, could have relevance in explaining the differential effects seen by the ODC1 SNP between neuroblastoma and adult cancers.

CREB1 is involved in tumorigenic processes such as proliferation, invasion and metastasis [37,38] and is overexpressed in many cancer types including neuroblastoma [39,40,41]. It has recently been identified as a direct target of miR-205 in neuroblastoma and colon cancer, with miR-205 playing a role in inhibiting CREB1 [42,43]. WT1 has been shown to be overexpressed and associated with poor outcome in several human tumors [44,45], although some reports suggest that WT1 is not associated with oncogenicity in neuroblastoma [46,47]. A recent study has found that WT1 expression is inversely correlated with MYCN expression in neuroblastoma, confirming our findings in this study, and whilst a mechanistic link is unclear, an association between high WT1 expression and poor outcome in non-MYCN amplified neuroblastomas was observed [48].

While the patient samples in our study cohort that expressed the highest levels of *ODC1* transcript exhibited a GG genotype, we were unable to show a significant difference in *ODC1* expression level in the neuroblastoma cohorts split by genotype. Thus, despite showing that the AG/GG genotypes lead to higher *ODC1* transcript levels compared to the AA genotype in vitro, the difficulty in confirming this in primary tumor samples may be linked to a number of factors including: the relatively rare frequency of the A allele; tumor/stromal cell heterogeneity; and other determinants such as epigenetic modifications (at the level of DNA and/or chromatin) that can heavily affect *ODC1* transcription. In addition, it is very difficult to measure ODC1 protein expression levels in tumor samples since ODC1 is a very low abundance protein and is highly regulated not only at the transcriptional level, but also by degradation. Interestingly, the SNP is embedded in a long CpG island in which CpG motifs are near each E-box, raising the possibility that the methylation status in this region may also influence the regulation of *ODC1* expression.

## 4. Materials and Methods

### 4.1. Cell Culture and Reagents

Tet21N cells, derived from the SHEP neuroblastoma cell line, stably express MYCN under the control of a tetracycline responsive promoter, where addition of tetracycline at 2 µg/mL represses expression of MYCN (Tet-off) [4]. Human neuroblastoma SK-N-BE(2)-C cells and Tet21N cells were maintained in Dulbecco’s Modified Eagle’s Medium (DMEM) (Life Technologies, Carlsbad, CA, USA) with 10% fetal calf serum (FCS) (Life Technologies, Carlsbad, CA, USA) at 37 °C/5% CO_2_. Their identities were verified by STR profiling (BMR Genomics, Padula, Italy) and cells were routinely mycoplasma tested.

### 4.2. CRISPR-Cas9 Genome Editing

Two specific single-guide RNAs (sgRNAs) targeting the G SNP were designed using the BlueHeron Guide RNA Target Design Tool (Blue Heron Biotech, Bothell, WA, USA), and CasOT 1.0 program (PKU Zebrafish Functional Genomics Group, Peking University, Beijing, China) [49]: sgRNA1: 5′-CGCCGGCCTGCGGAGACACG-3′ and sgRNA2: 5′-CGGCGACCACGTGTCTCCGC-3′. The sgRNAs were cloned into the pCas-Guide-EF1a-GFP vector (Origene, Rockville, MD, USA) and transfected into SK-N-BE(2)-C cells. Cleavage efficiencies were tested using GeneArt Genomic Cleavage Detection Kit (Thermo Fisher Scientific, Waltham, MA, USA) and sgRNA1 was selected for subsequent experiments. The absence of off-target activity was verified on the most probable off-target site by GeneArt Genomic Cleavage Detection Kit and confirmed by DNA sequencing. A donor template DNA was employed to mediate the A/G switch of the SNP by homology-directed repair (HDR). Donor DNA was amplified by PCR from NBL-S genomic DNA (primers: 5′-GTGCTATAAGTAGGGAGCG-3′ and 5′-AAACTGGAAGGAAACTGAAG-3′). The obtained donor DNA was 683 bp-long with 351 bp and 331 bp homologous arms. SK-N-BE(2)-C cells were transfected using Lipofectamine 3000 (Thermo Fisher Scientific, Waltham, MA, USA), and isolated by serial dilutions 48 h later. Clonal cell lines were picked, expanded and characterized by RFLP analysis and DNA sequencing. Of the 81 screened clones, the calculated efficiency of successful heterozygous HDR-editing was 2.5%, while 16% carried indel mutations.

### 4.3. BrdU Cell Proliferation Assay

Proliferation rates of CRISPR-edited SK-N-BE(2)-C clones were measured using bromodeoxyUridine (BrdU) incorporation (Cell Proliferation ELISA, BrdU (colorimetric), Roche, Basel, Switzerland), according to manufacturer’s instructions. Cells were cultured for 7 days. 10 μM BrdU was added and incorporation of BrdU quantified by ELISA 24 h later.

### 4.4. ODC1 Expression in CRISPR-Edited Clones

ODC1 expression in CRISPR-edited clones was determined by qRT-PCR and Western blot as previously described [50]. Primers are listed in Table S5. Rabbit monoclonal anti-Odc1 (Abcam, Cambridge, UK; ab126590) and rabbit monoclonal anti-β-Actin (Sigma-Aldrich, St. Louis, MO, USA; a2066) antibodies were used.

### 4.5. Chromatin-Immunoprecipitation (ChIP)

Standard ChIP assays were performed as previously described [50]. The analyzed region is shown in Figure S3A. Anti-Acetyl-Histone H3 (Merck, Rahway, NJ, USA; 06-599) antibody was used and primers are listed in Table S5.

### 4.6. Clonogenic Assay

Colony assays were performed as previously described [13]. SK-N-BE(2)-C cells were treated with 0–0.4 mM DFMO (Vinci Biochem, Florence, Italy). The total surface area occupied by the colonies within each well was determined using ImageJ software (version 1.51, NIH and LOCI, Madison, WI, USA).

### 4.7. Electrophoretic Mobility Shift Assays (EMSA)

SNP A/G DNA minimal probes, corresponding to region +301 to +334 of the *ODC1* promoter including SNP A/G, were prepared from single strand synthetic oligonucleotides (Table S6). DNA probes used for cold competition assays were prepared from synthetic oligonucleotides containing the canonical wild type E-box using previously validated probes [51] and the mutated E-box (Table S6). Constructs are shown in Figure S3B. Complementary sense and antisense oligonucleotides were annealed, double stranded probes were 5′ termini labelled with [γ^−32^P]ATP using T4 Polynucleotide Kinase (New England Biolabs, MA, USA) and labelled probes were purified using QIAquick Nucleotide Removal Kit (Qiagen, Hilden, Germany). Nuclear protein extracts were prepared from SK-N-BE(2)-C and Tet21N cell lines as previously described [52]. Binding reactions were performed in EMSA-binding buffer using 100 fmol of each labelled probe and 10 µg of nuclear protein extract at 20 °C for 35 min. For competition experiments, 5-, 10- and 25-fold molar excess of wildtype or mutated unlabeled DNA probes were added. The reaction mixture was separated on 5% polyacrylamide gel then exposed to Kodak^®^ BioMax^®^ MS film (Kodak, Rochester, NY, USA) overnight at −80 °C. The relative intensities of the bands (normalized for each specific free probe stain) were analyzed with Bio-Rad Quantity One^®^ 1-D Analysis software (version 25.0, Bio-Rad Laboratories, Hercules, CA, USA).

### 4.8. Luciferase Reporter Assay

The *ODC1* promoter region containing the SNP G316A (from +257 to +330) was amplified by nested PCR from SK-N-BE(2)-C (GG genotype) and NBL-S (AA genotype) genomic DNA. The sequences containing SNP G and SNP A were cloned into the luciferase pGL3 basic promoter vector (Promega, Fitchburg, WI, USA) (construct used is shown in Figure S3B). The Renilla-TK vector was used as an internal control. Firefly or Renilla luciferase activity was measured with the Dual Luciferase Assay kit (Promega).

### 4.9. Cohort Descriptions

Our study cohort is made up of neuroblastoma samples from the USA, Australia and Europe. The USA samples are from 183 patients enrolled between 1994–1998 by the Pediatric Oncology Group [53]. Australian samples are from 185 patients who were diagnosed in Australia and New Zealand between 1985–2000 [54]. DNA for the USA and Australian samples was isolated and RNA extracted as previously described [53,55,56]. The European samples are made up of 148 tumor RNA samples from the International Society of Pediatric Oncology European Neuroblastoma Group study (SIOPEN) [57], and 142 tumor RNA samples from the European Neuroblastoma Research Consortium (NRC), collected before 2012. DNA was amplified prior to genotyping as previously described [58]. RNA was available and of sufficient quality for expression analysis for 290 tumors.

GWAS cohort: Peripheral blood or uninvolved bone marrow DNA from 4892 neuroblastoma cases diagnosed up until 2016 in the USA and registered through the Children’s Oncology Group (COG) was genotyped using Illumina HumanHap550 v1, HumanHap550 v3, HumanHap550 v3duo, Human610 Quad v1.0 and OmniExpress-24 SNP arrays, as described previously [59]. Genotypes for this multi-ethnic cohort were merged using PLINK [60] (v1.90p) and haplotypes inferred using SHAPEIT [61] (v2.r881). To impute genotypes across the genome, we utilized IMPUTE2 [62] (v2.3.2) with default parameters and Ne = 20000, along with a multi-population reference panel from the world-wide 1000 Genomes Project Phase 3. Imputed genotypes for rs2302615 were required to achieve an IMPUTE2 info quality score >0.9 and have inferred genotype probability ≥0.90.

Lung Cancer cohort: DNA was extracted from fresh frozen surgically resected tumor tissue obtained from 366 patients with non-small cell lung cancer (NSCLC) who donated remnant tissue samples to The Prince Charles Hospital, Brisbane, Australia between 1992–2009. The cohort included 169 patients with adenocarcinoma, 161 patients with squamous cell carcinoma (SCC) and 36 patients with other histologies. The study was approved by the human research ethics committees at The Prince Charles Hospital (HREC EC9124, 17 October 2008) and Sydney Children’s Hospital, part of the South Eastern Sydney Area Health Service (07/101, 22 May 2007—replaced by The University of New South Wales (HC12551, 16 October 2012).

### 4.10. ODC1 Expression in Neuroblastoma Patients

For the USA samples of the study cohort, RNA was reverse-transcribed using MMLV reverse-transcriptase (Life Technologies). Gene expression levels were quantified by qRT-PCR using TaqMan^®^ Gene Expression Assays (Applied Biosystems™, Foster City, CA, USA) on a 96 × 96 Integrated Fluidics Circuit (BioMark HD System, Fluidigm, San Francisco, CA, USA). Data was normalized to a panel of control genes, *HPRT1*, *GUSB*, *PPIA*, *HMBS* and *SDHA*. Taqman assays used were *ODC1* Hs00159739_m1, *HPRT1* Hs99999909_m1, *GUSB* Hs99999908_m1, *PPIA* Hs99999904_m1, *HMBS* Hs00609296_g and *SDHA* Hs00188166_m1.

For the European samples of the study cohort, qRT-PCR based analysis of gene expression on a LightCycler480 (Roche) was performed as previously described [57]. *ODC1* expression was measured using a previously reported TaqMan assay [10]. Measurement of Alu-Sq, *HMBS*, *HPRT1*, *SDHA* and *UBC* expression and normalization of *ODC1* expression using the geometric mean expression levels of these five reference sequences were performed as previously described [58].

### 4.11. SNP Genotyping

RT-PCR was used to genotype the *ODC1* promoter SNP G316A [14]. Primers and probes specific for either the G or A allele were designed and supplied by Applied Biosystems Custom Taqman^®^ SNP Genotyping Assay Service (Barcode 0039116973, Applied Biosystems, Foster City, CA, USA). The minor groove binder fluorogenic probes were labelled with FAM or VIC specific for the G and A alleles, respectively. Primer and probe sequences were: ODC-G316A-F 5′-CCGGGCACGTGTGC-3′; ODC-G316A-R 5′-GAAGCGGCGCCTCAAG-3′; ODC-G probe 5′-CTGCGGAGACACG-3′; ODC-A probe 5′-CCTGCAGAGACACG-3′. 2x TaqMan Genotyping Master Mix (Applied Biosystems) and 40× ODC1 primer/probes mix was added to 10 ng of genomic DNA. Reactions were subjected to a 10-min denaturation at 95 °C, followed by 40 cycles of two-step PCR (92 °C for 15 s, 60 °C for 1 min). A post-read was immediately performed using the ABI Prism 7900 sequence detection system to determine endpoint fluorescence. PCR reactions were performed in duplicate and allelic discrimination was determined by laboratory personnel blinded to clinical data.

### 4.12. Statistical Procedures

Associations between clinical characteristics of patients and molecular characteristics of tumors were examined using Fisher’s exact test. Associations with event-free survival (EFS) and overall survival (OS) were determined using Kaplan-Meier analyses and log-rank tests, or Cox regression for multivariate analyses, using SPSS version 24 (IBM, Armonk, NY, USA) as previously described [63]. For expression analyses, tumors were categorized as having high or low gene expression based on median cut-points, and a Cox model produced a *p* value and a hazard ratio.

Experiments were repeated at least 3 times and the mean ± standard error calculated. Differences between 2 groups were determined with an unpaired two-tailed Student’s *t*-test. For 3 groups, one-way ANOVA tests were performed with correction according to Giesser-Greenhouse. To examine the influence of the SNP (A versus G) and MYCN expression (not expressed versus overexpressed) on promoter activity, a two-way ANOVA was conducted followed by Tukey’s post-hoc tests for multiple pairwise comparisons. A probability level *p* < 0.05 was considered to be statistically significant.

To identify transcriptional regulators of ODC1 expression, the DoRothEA interactions dataset, which is available through the OmniPath package and Bioconductor software in RStudio (version 1.2.1335, R Core Team, Vienna, Austria), was utilized. Only transcription factors with a high confidence level (rated ‘A’), derived from the number of supporting evidences of a TF-interaction, were selected. These were then explored using the MegaSamper module in the R2 Analysis Genomics and Visualization platform where datasets that use the same chip (u133p2) and the same normalization method (MAS5.0) can be compared. A one-way ANOVA was used to detect significant differences across the datasets, and if a significant difference was detected Tukey’s post-hoc tests for multiple pairwise comparisons were performed.

## 5. Conclusions

We have shown that the *ODC1* G316A polymorphism is functionally important in childhood neuroblastoma. In particular, we found allele-specific regulation of this SNP by MYCN, in which this oncoprotein preferentially binds the G allele over the A allele leading to a greater stimulatory effect on the *ODC1* promoter. These results in neuroblastoma contrast with those in adult cancers, and suggest tumor-specific regulation of *ODC1* by E-box transcription factors.

## Figures and Tables

**Table 1:** The clinical and molecular characteristics of the 839 patients of the study cohort, and the 4892 patients of the GWAS cohort. In the study cohort, age was available for 638 patients, stage was available for 799 patients and *MYCN* amplification status was available for 837 patients. In the GWAS cohort, age was available for all patients, risk group was available for 4787 patients and *MYCN* amplification status was available for 4539 patients.

Factors			Genotype			Total	*p*
			AA	AG	GG		
Study cohort	Age	≤18 months	36 (7.9%)	141 (31.0%)	278 (61.1%)	455	0.551
		>18 months	25 (6.5%)	130 (33.9%)	228 (59.5%)	383	
	Tumor stage	Favourable	31 (8.9%)	96 (27.5%)	223 (63.7%)	350	0.049
		Unfavourable	28 (6.2%)	158 (35.2%)	263 (58.6%)	449	
	*MYCN* status	Non-amplified	50 (7.2%)	224 (32.2%)	421 (60.6%)	695	0.970
		Amplified	11 (7.7%)	45 (31.7%)	86 (60.6%)	142	
GWAS cohort	Age	≤18 months	187 (8.3%)	887 (39.4%)	1177 (52.3%)	2251	0.328
		>18 months	238 (9.0%)	1076 (40.7%)	1327 (50.2%)	2641	
	Risk group	Low	144 (9.2%)	622 (39.9%)	794 (50.9%)	1560	0.826
		Intermediate	100 (8.8%)	451 (39.9%)	580 (51.3%)	1131	
		High	171 (8.2%)	855 (40.8%)	1070 (51.0%)	2096	
	*MYCN* status	Non-amplified	341 (9.2%)	1469 (39.7%)	1889 (51.1%)	3699	0.033
		Amplified	54 (6.4%)	349 (41.5%)	437 (52.0%)	840	

**Table 2:** Univariate Cox regression analysis of *ODC* SNP genotype groupings in neuroblastoma patients of the study cohort (EFS available for 839 neuroblastoma patients, and OS for 838), and the GWAS cohort (EFS and OS available for all patients).

Sample	Genotype	Event-Free Survival		Overall Survival	
		Relative Hazard (95% CI)	*p*	Relative Hazard (95% CI)	*p*
Study cohort					
All patients	GG	0.98 (0.77–1.24)	0.847	0.99 (0.75–1.29)	0.914
	AG/GG	1.63 (0.95–2.78)	0.074	1.45 (0.81–2.59)	0.212
MYCN non-amplified	GG	0.79 (0.59–1.06)	0.120	0.76 (0.54–1.07)	0.115
	AG/GG	2.46 (1.09–5.45)	0.025	2.05 (0.84–5.01)	0.103
MYCN amplified	GG	1.93 (1.27–2.93)	0.001	1.91 (1.22–2.97)	0.003
	AG/GG	1.07 (0.52–2.21)	0.846	1.13 (0.52–2.45)	0.752
GWAS cohort					
All patients	GG	0.96 (0.87–1.07)	0.469	0.98 (0.87–1.11)	0.792
	AG/GG	1.10 (0.91–1.32)	0.323	1.14 (0.92–1.43)	0.237
MYCN non-amplified	GG	0.88 (0.77–1.01)	0.063	0.87 (0.74–1.03)	0.107
	AG/GG	1.02 (0.81–1.29)	0.858	1.03 (0.78–1.37)	0.828
MYCN amplified	GG	1.13 (0.94–1.36)	0.197	1.15 (0.95–1.40)	0.152
	AG/GG	0.93 (0.65–1.34)	0.711	1.12 (0.74–1.69)	0.597

Full multivariate analysis is shown in Table S2.

## Data Availability

The data for the study cohort is available on request from the authors. The data for the GWAS cohort is available through the Database of Genotypes and Phenotypes (dbGaP) (Accession phs000124, https://www.ncbi.nlm.nih.gov/projects/gap/cgi-bin/study.cgi?study_id=phs000124.v3.p1).

## Supplementary Materials

The following are available online at https://www.mdpi.com/article/10.3390/cancers13081807/s1, Table S1: Our study cohort is made up of samples from Europe, the USA and Australia, Table S2: Multivariate analyses for the different genotypes in the 839 patient study cohort (EFS data is available for 839 patients and OS data for 838 patients), and in the 4892 patient GWAS cohort (EFS and OS is available for all patients), Table S3: The number of each genotype in a non-small cell lung cancer cohort of 366 patients of mixed histologies (all), and in split cohorts of adenocarcinoma and squamous cell carcinoma, and the prognostic impact of these genotypes on outcome, Table S4: Multivariate analyses for the squamous cell carcinoma cohort (161 patients), Table S5: Primers used for qRT-PCR analysis of *ODC1* expression (rows 1–4) and for ChIP (rows 5–16) in CRISPR-edited clones, Table S6: Probes used for EMSA assays, Figure S1: The ODC1 SNP at +316 is in intron 1 of the ODC1 transcript, and lies between 2 consensus E-box binding elements, Figure S2: Sequencing of the parental SK-N-BE(2)-C cells which are of GG genotype at the +316 SNP site, and the two AG clones generated by CRISPR-Cas9 technology, Figure S3: Schematic diagrams of the structure of the *ODC1* gene and promoter, Figure S4: Quantification of the EMSA assays shown in Figure 2A, Figure S5: Survival analysis stratified by stage/risk group, age and *MYCN* amplification status, Figure S6: Survival analysis for the study cohort and the GWAS cohort, Figure S7: Separate survival analysis of the three distinct cohorts that were combined to make the study cohort, Figure S8: Survival analysis for high-risk patients, Figure S9: Expression of the transcriptional regulators of ODC1, as identified via the DoRothEA interactions dataset available in OmniPath, across the multiple tumor types, Figure S10: Uncropped WB of Figure 1C.
