# Supplementary material for: A G316A Polymorphism in the Ornithine Decarboxylase Gene Promoter Modulates MYCN-Driven Childhood Neuroblastoma
Source: Cancers (Basel). 2021 Apr 9;13(8):1807. doi: 10.3390/cancers13081807 (PMC8069650; doi:10.3390/cancers13081807)
Supplement: Supplementary file 1 [file cancers-13-01807-s001.pdf]

Supplementary Material: A G316A Polymorphism in the Ornithine Decarboxylase Gene Promoter Modulates MYCN-Driven Childhood Neuroblastoma

Laura D. Gamble, Stefania Purgato, Michelle J. Henderson, Simone Di Giacomo, Amanda J. Russell, Paolo Pigini, Jayne Murray, Emanuele Valli, Giorgio Milazzo, Federico M. Giorgi, Mark Cowley, Lesley J. Ashton, Jaydutt Bhalshankar, Gudrun Schleiermacher, Ali Rihani, Tom Van Maerken, Jo Vandesompele, Frank Speleman, Rogier Versteeg, Jan Koster, Angelika Eggert, Rosa Noguera, Raymond L. Stallings, Gian Paolo Tonini, Kwun Fong, Zalman Vaksman, Sharon J. Diskin, John M. Maris, Wendy B. London, Glenn M. Marshall, David S. Ziegler, Michael D. Hogarty, Giovanni Perini, Murray D. Norris and Michelle Haber

Table S1. Our study cohort is made up of samples from Europe, the USA and Australia. We also have a large GWAS cohort which includes samples from more recently diagnosed neuroblastoma patients. The proportion of patients with each of the three genotypes AA, AG and GG are displayed.

Cohort	AA (%)	AG (%)	GG (%)	Total
Overall cohort	61 (7.3)	272 (32.4)	506 (60.3)	839
European samples	35 (7.4)	124 (26.3)	312 (66.3)	471
USA samples	14 (7.6)	77 (42.1)	92 (50.3)	183
Australian samples	12 (6.5)	71 (38.4)	102 (55.1)	185
GWAS cohort	425 (8.7)	1963 (40.1)	2504 (51.2)	4892

Table S2. Multivariate analyses for the different genotypes in the 839 patient study cohort (EFS data is available for 839 patients and OS data for 838 patients), and in the 4892 patient GWAS cohort (EFS and OS is available for all patients). Cox proportional hazards analysis adjusted for *MYCN* status (amplified vs. non-amplified), stage (favourable vs. unfavourable) or risk group (low/intermediate vs. high), and age at diagnosis (≤18 months vs. >18 months). In the *MYCN* amplified patients of the GWAS cohort, only 2.5% of patients were not high risk so this was not adjusted for due to small sample size.

Study Cohort	Factor	Event-Free Survival (n = 839)		Overall Survival (n = 838)	
		Relative Hazard (95% CI)	p	Relative Hazard (95% CI)	p
All patients	GG genotype	1.18 (0.92–1.52)	0.186	1.24 (0.94–1.65)	0.132
	Unfavourable stage	5.37 (3.58–8.05)	<0.001	13.84 (6.99–27.38)	<0.001
	Older age	2.16 (1.62–2.88)	<0.001	2.35 (1.67–3.29)	<0.001
	MYCN amplification	2.30 (1.76–3.01)	<0.001	2.65 (1.98–3.56)	<0.001
	AG/GG genotype	1.41 (0.82–2.41)	0.216	0.79 (0.44–1.42)	0.431
	Unfavourable stage	5.29 (3.53–7.94)	<0.001	13.66 (6.90–27.05)	<0.001
	Older age	2.14 (1.61–2.86)	<0.001	2.33 (1.66–3.27)	<0.001
	MYCN amplification	2.29 (1.75–2.99)	<0.001	2.61 (1.95–3.50)	<0.001
MYCN non-amplified	GG genotype	0.88 (0.65–1.20)	0.421	0.83 (0.58–1.18)	0.292
	Unfavourable stage	5.46 (3.50–8.52)	<0.001	13.22 (6.10–28.64)	<0.001
	Older age	3.26 (2.29–4.63)	<0.001	5.01 (3.14–8.00)	<0.001
	AG/GG genotype	1.75 (0.77–3.97)	0.178	1.31 (0.53–3.21)	0.558
	Unfavourable stage	5.39 (3.45–8.41)	<0.001	13.16 (6.07–28.53)	<0.001
	Older age	3.12 (2.27–4.59)	<0.001	4.96 (3.10–7.92)	<0.001
MYCN amplified	GG genotype	1.85 (1.19–2.88)	0.006	1.94 (1.21–3.11)	0.006
	Unfavourable stage	2.74 (1.10–6.82)	0.031	7.92 (1.93–32.51)	0.004
	Older age	0.84 (0.53–1.34)	0.476	0.71 (0.44–1.14)	0.153
	AG/GG genotype	1.06 (0.51–2.19)	0.880	1.14 (0.52–2.48)	0.741
	Unfavourable stage	2.56 (1.03–6.37)	0.043	7.14 (1.74–29.29)	0.006
	Older age	0.79 (0.50–1.26)	0.317	0.65 (0.40–1.04)	0.075
**GWAS Cohort**	**Factor**	**Event-Free Survival (n = 4892)**		**Overall Survival (n = 4892)**	
		**Relative Hazard (95% CI)**	p	**Relative Hazard (95% CI)**	p
All patients	GG genotype	0.97 (0.87–1.08)	0.572	1.00 (0.89–1.14)	0.944
	High risk	4.91 (4.19–5.76)	<0.001	10.70 (8.48–13.51)	<0.001
	Older age	1.07 (0.93–1.23)	0.355	1.23 (1.03–1.46)	0.021

	MYCN amplification	1.22 (1.07–1.38)	0.003	1.45 (1.26–1.67)	<0.001
	AG/GG genotype	1.01 (0.83–1.23)	0.923	1.07 (0.84–1.35)	0.602
	High risk	4.91 (4.19–5.77)	<0.001	10.70 (8.48–13.51)	<0.001
	Older age	1.07 (0.93–1.23)	0.354	1.23 (1.03–1.46)	0.020
	MYCN amplification	1.22 (1.07–1.38)	0.003	1.45 (1.26–1.67)	<0.001
MYCN non-amplified	GG genotype	0.90 (0.79–1.03)	0.115	0.92 (0.77–1.08)	0.298
	High risk	4.78 (3.99–5.72)	<0.001	8.65 (6.67–11.22)	<0.001
	Older age	1.12 (0.93–1.36)	0.235	1.85 (1.37–2.49)	<0.001
	AG/GG genotype	1.07 (0.85–1.34)	0.581	1.08 (0.81–1.44)	0.582
	High risk	4.77 (3.99–5.71)	<0.001	8.65 (6.67–11.22)	<0.001
	Older age	1.13 (0.93–1.37)	0.219	1.86 (1.38–2.50)	<0.001
MYCN amplified	GG genotype	1.13 (0.94–1.36)	0.199	1.15 (0.95–1.40)	0.152
	Older age	1.02 (0.84–1.24)	0.846	1.00 (0.81–1.23)	1.000
	AG/GG genotype	0.93 (0.65–1.35)	0.439	1.12 (0.74–1.69)	0.597
	Older age	1.02 (0.84–1.25)	0.831	1.00. (0.81–1.24)	0.990

Table S3. The number of each genotype in a non-small cell lung cancer cohort of 366 patients of mixed histologies (all), and in split cohorts of adenocarcinoma and squamous cell carcinoma, and the prognostic impact of these genotypes on outcome.

**Genotype**	**Non-Small Cell Lung Cancer**		
	**All**	**Adenocarcinoma**	**Squamous Cell Carcinoma**
AA	22	11	11
AG	157	55	73
GG	187	103	77
AA vs. AG/GG	*p* = NS	*p* = NS	*p* = NS
AA/AG vs. GG	*p* = 0.039	*p* = NS	*p* = 0.017

NS: not significant.

Table S4. Multivariate analyses for the squamous cell carcinoma cohort (161 patients). Cox proportional hazards analysis adjusted for *ECOG status* (0 vs. 1–2), stage (1–2 vs. 3–4), and age at diagnosis.

**Multivariate Analysis Of 161 SCC Lung Cancer Patients**		
**Factor**	**Overall Survival**	
	**Relative Hazard (95% Confidence Interval)**	***p*-value**
Genotype AA/AG vs. GG	1.45 (1.01–2.09)	0.049
ECOG status 0 vs. 1–2	1.60 (1.10–2.34)	0.014
Stage 1/2 vs. 3/4	2.62 (1.57–4.34)	<0.001
Age	1.03 (1.01–1.05)	0.010

Table S5. Primers used for qRT-PCR analysis of *ODC1* expression (rows 1–4) and for ChIP (rows 5–16) in CRISPR-edited clones.

**Genomic Target**	**Sequence**
ODC1 Fw	TGCTGCCTCTACGTTCAATG
ODC1 Rv	GTTCTGGAATTGCTGCATGA
GUSB Fw	AGCCTGGAGCAAGACAGTGG
GUSB Rv	ATACAGATAGGCAGGGCGTTCG
−15000 bp (forward)	AGACTCTCCCTGGCCAAGAT
−15000 bp (reverse)	AGCTCTCACCTCCAGATTGC
E-box 1 (forward)	ATCACTTCCAGGTCCCTTGC

E-box 1 (reverse)	GAGAGCGGAAAAGGGAAATC
+316 A/G SNP (forward)	TTCTGCCCCCGTCTTCACAG
+316 A/G SNP (reverse)	CCGAAGGGTTGGGAAAGAGG
Exon 9 (forward)	AATCAACCCAGCGTTGGACA
Exon 9 (reverse)	CAGAGCCCGTCTGTTCCTTT
+1500 bp (forward)	AAGGGCCAAGGAAGATCACT
+1500 bp (reverse)	CTGAAACCTCGCTTCTGACC
β-actin (forward)	GCAGAAGAGAGAACCAGTGAGAA
β-actin (reverse)	GAGAAGATGACCCAGGTGAGTG

Table S6. Probes used for EMSA assays.

Probe	Sequence
SNP-G sense	5′_GCCTCGCCGGCCTGC**G**GAGA**CACGTG**GTCGCCGA_3′
SNP-G antisense	5′_TCGGCGAC**CACGTG**TCTC**C**GCAGGCCGGCGAGGC_3′
SNP-A sense	5′_GCCTCGCCGGCCTGC**A**GAGA**CACGTG**GTCGCCGA_3′
SNP-A antisense	5′_TCGGCGAC**CACGTG**TCTC**T**GCAGGCCGGCGAGGC_3′
WT E-Box sense	5′-CGGCAGCGAGC**CACGTG**GACCAACTACCT-3′
WT E-box antisense	5′-AGGTAGTTGGTC**CACGTG**GCTCGCTGCCG-3′
Mutated E-box sense	5′_CGGCAGCGAGC**ATCATC**ATCGACCAACTACCT_3′
Mutated E-box antisense	5′_AGGTAGTTGGTC**GATGAT**GCTCGCTGCCG_3′

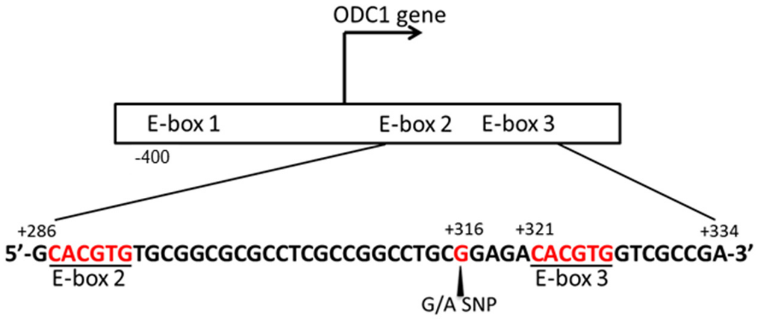

Figure S1. The ODC1 SNP at +316 is in intron 1 of the ODC1 transcript, and lies between 2 consensus E-box binding elements. The three resulting genotypes are wildtype GG, and variants AG and AA.

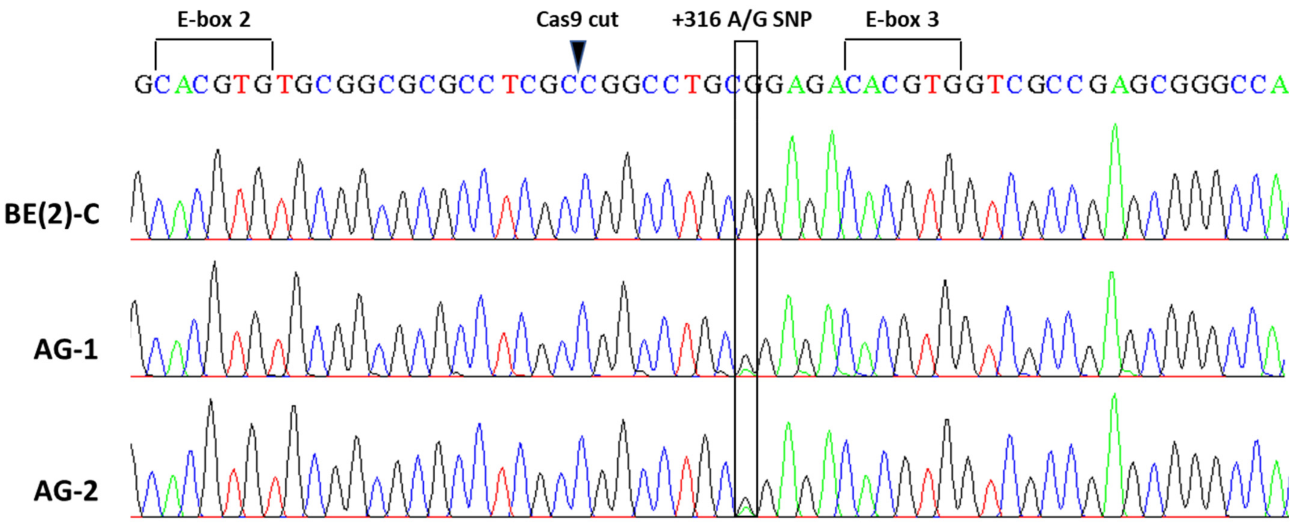

Figure S2. Sequencing of the parental SK-N-BE(2)-C cells which are of GG genotype at the +316 SNP site, and the two AG clones generated by CRISPR-Cas9 technology.

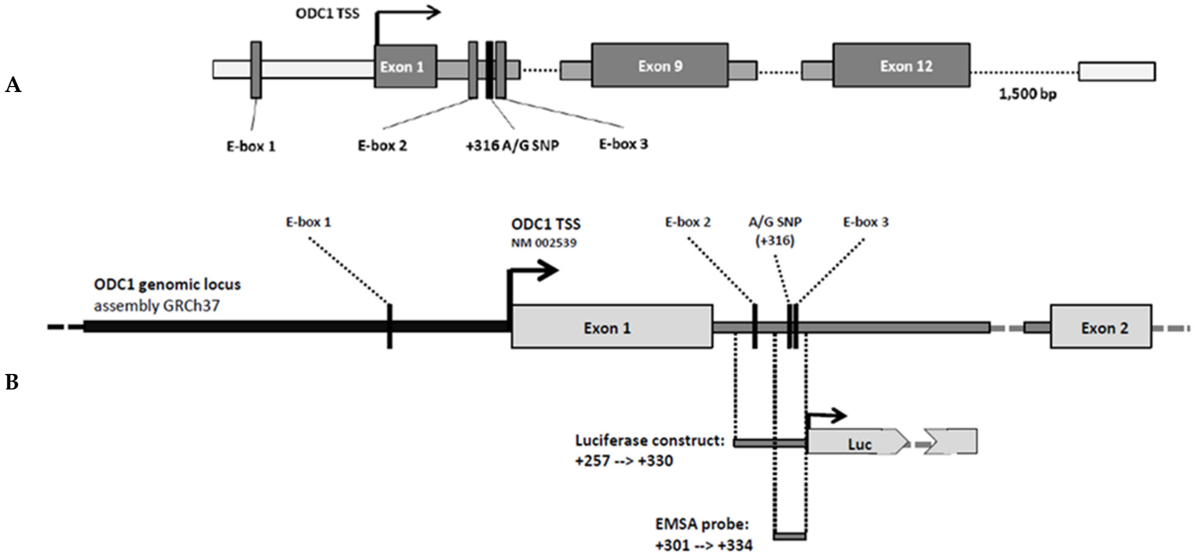

Figure S3. Schematic diagrams of the structure of the *ODC1* gene and promoter. (**A**) Region of ODC1 locus analysed by ChIP in CRISPR-edited clones. (**B**) Schematic representation of the constructs utilized for the luciferase reporter assay and EMSA.

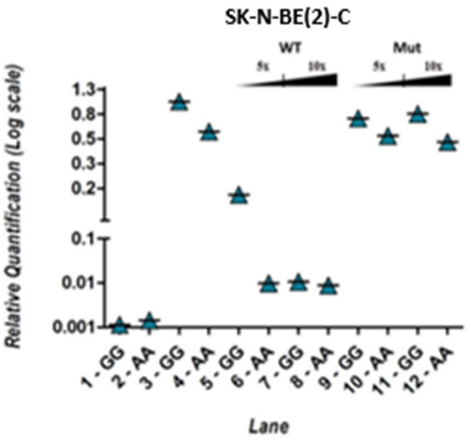

Normalised Relative Quantification	
Lane 1	0.001
Lane 2	0.001
Lane 3	1.000
Lane 4	0.573
Lane 5	0.178
Lane 6	0.010
Lane 7	0.011
Lane 8	0.009
Lane 9	0.733
Lane 10	0.531
Lane 11	0.799
Lane 12	0.474

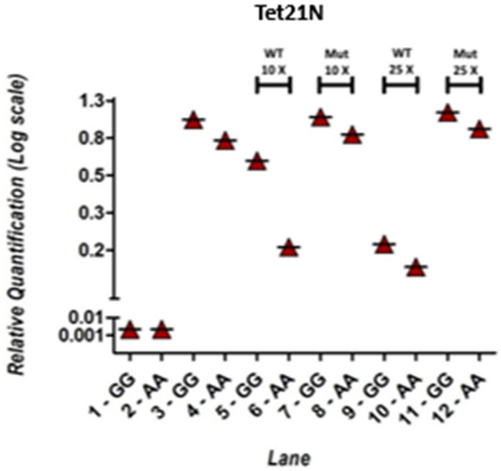

Normalised Relative Quantification	
Lane 1	0.002
Lane 2	0.002
Lane 3	1.000
Lane 4	0.774
Lane 5	0.602
Lane 6	0.208
Lane 7	1.031
Lane 8	0.831
Lane 9	0.215
Lane 10	0.163
Lane 11	1.093
Lane 12	0.891

Figure S4. Quantification of the EMSA assays shown in Figure 2A. Quantification was performed using Quantity one® 1-D Bio-Rad software.

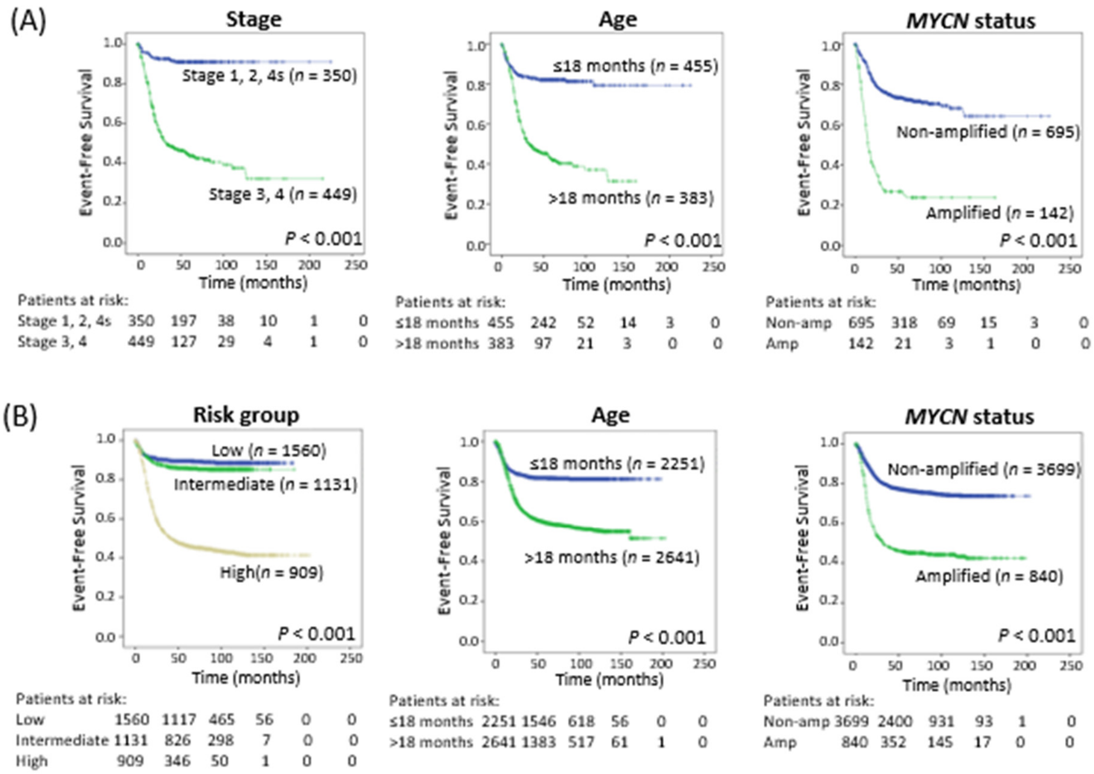

Figure S5. Survival analysis stratified by stage/risk group, age and *MYCN* amplification status. High stage (3 and 4), age (>18 months), and *MYCN* amplification are prognostic of poor event-free survival in the study cohort of neuroblastoma patients (n = 839) (**A**), and high risk group, age (> 18 months) and *MYCN* amplification are prognostic of poor event-free survival in the GWAS cohort (n = 4892) (**B**).

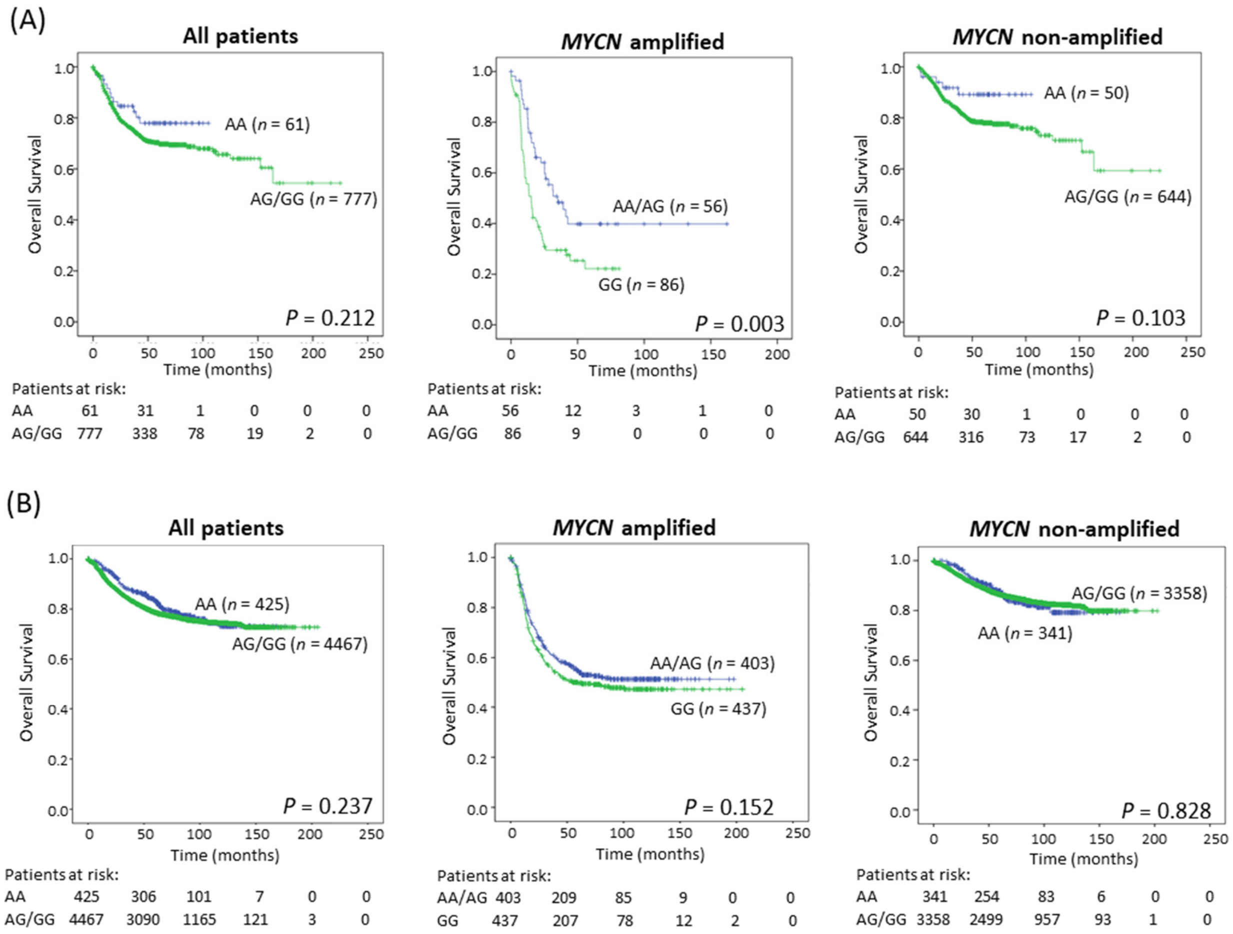

Figure S6. Survival analysis for the study cohort and the GWAS cohort. Overall survival for the 838 neuroblastoma study cohort (**A**), and the 4892 patient GWAS cohort (**B**), grouped by genotype (AA vs AA/AG for all patients and *non-MYCN* amplified patients, and AA/AG vs. GG for *MYCN* amplified patients).

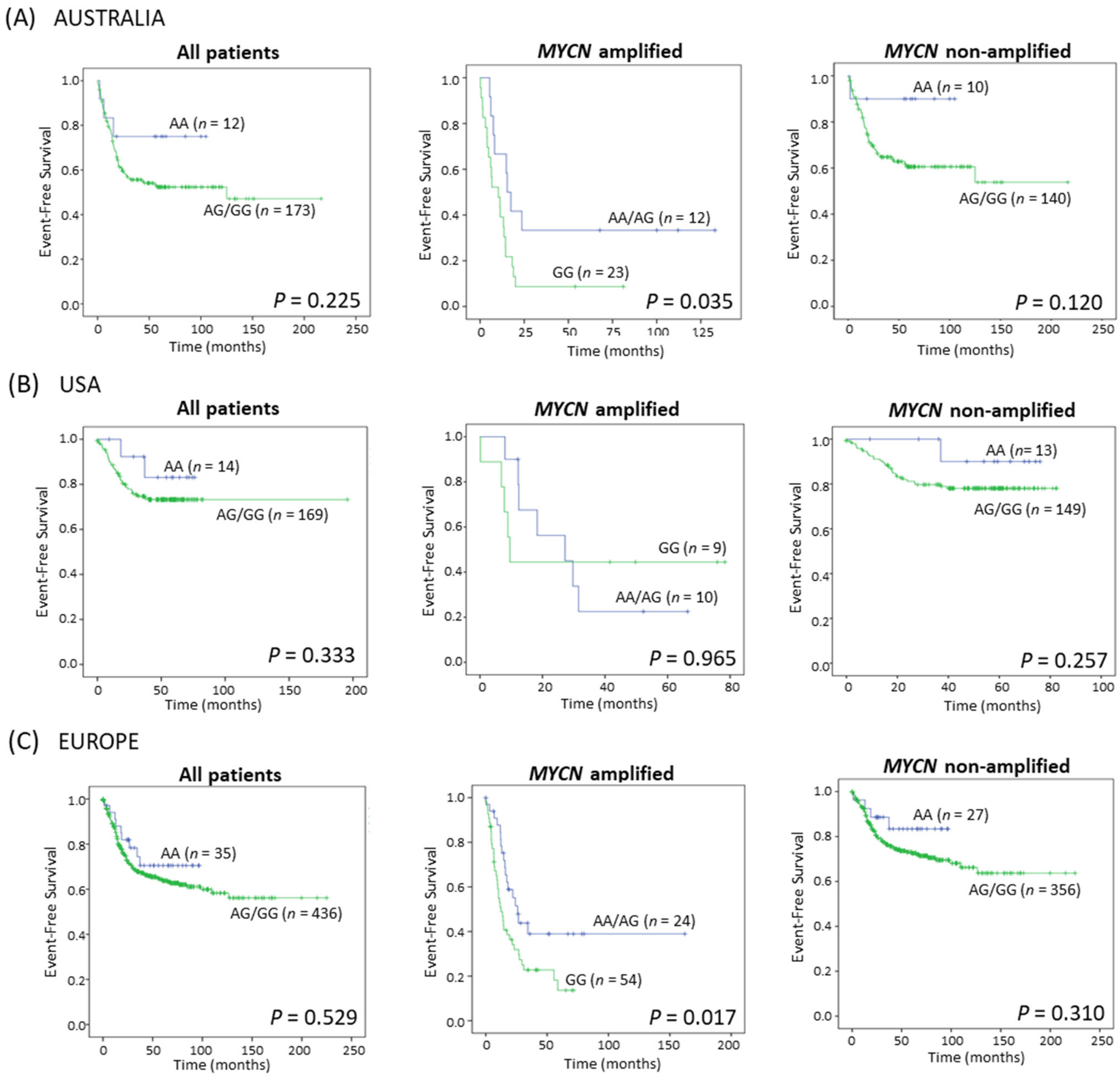

Figure S7. Separate survival analysis of the three distinct cohorts that were combined to make the study cohort. Australia (**A**), USA (**B**) and Europe (**C**).

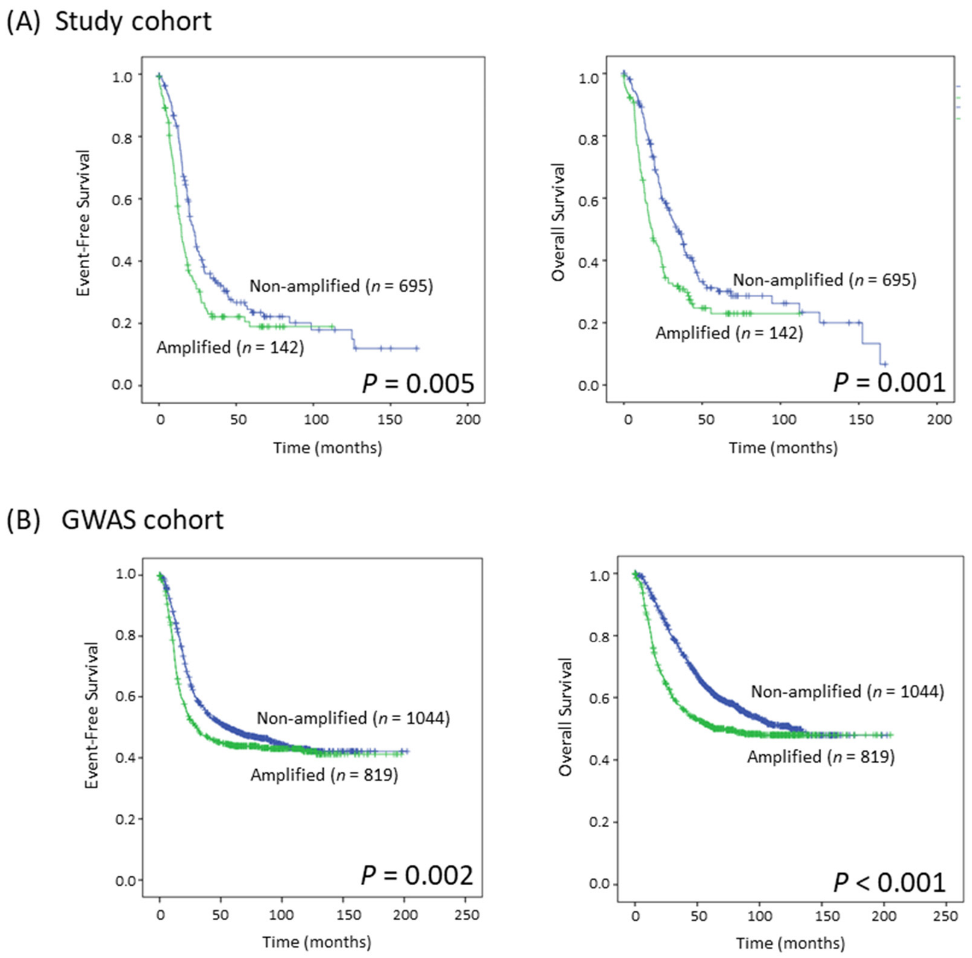

Figure S8. Survival analysis for high-risk patients**.** Event-free and overall survival for the high-risk patients of the study cohort (**A**) and the GWAS cohort (**B**), stratified by *MYCN* amplification status.

(A)

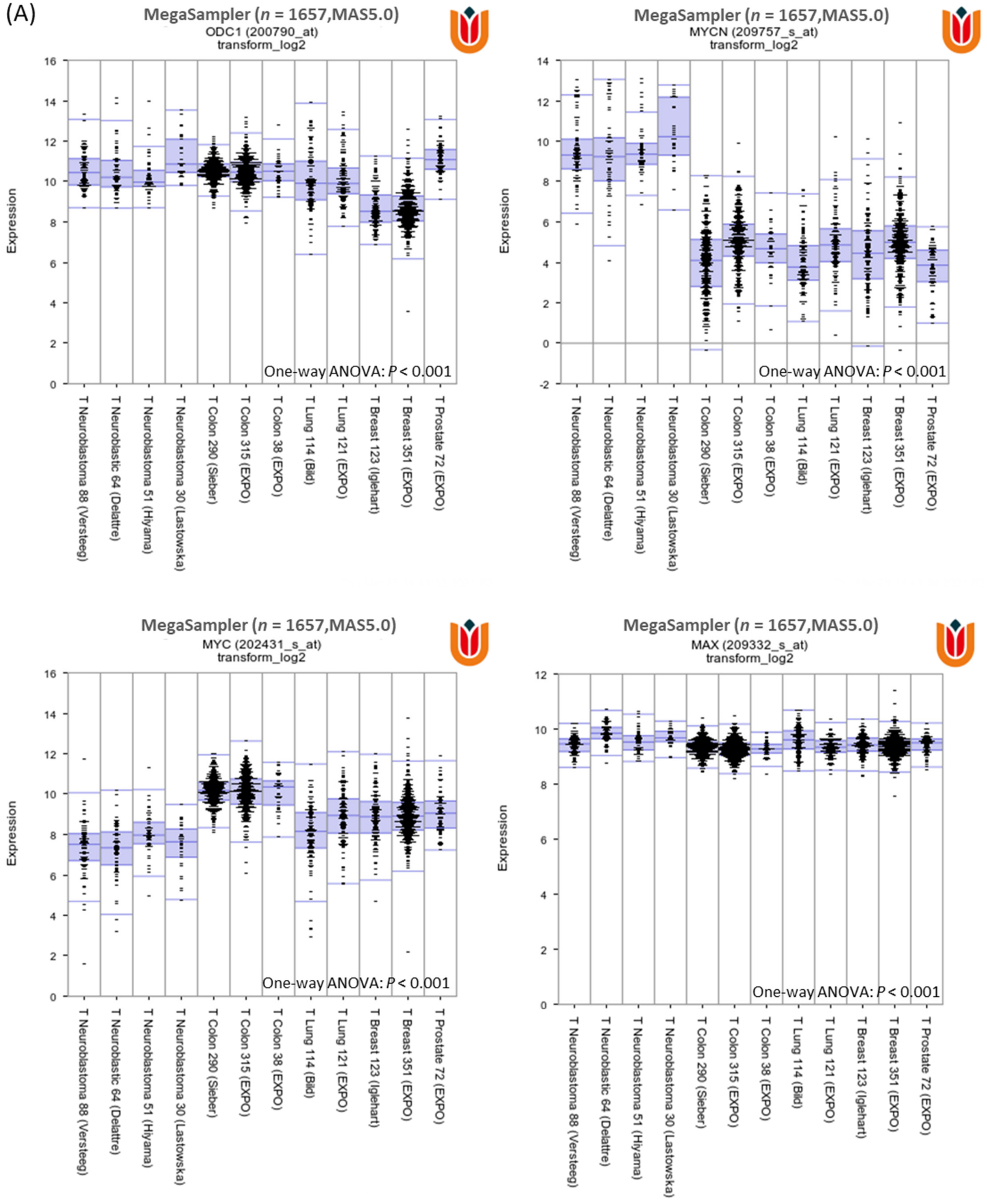
MegaSampler (*n* = 1657,MAS5.0)
ODC1 (200790_at)
transform_log2
MegaSampler (*n* = 1657,MAS5.0)
MYCN (209757_s_at)
transform_log2
MegaSampler (*n* = 1657,MAS5.0)
MYC (202431_s_at)
transform_log2
MegaSampler (*n* = 1657,MAS5.0)
MAX (209332_s_at)
transform_log2
Expression
One-way ANOVA: *P* < 0.001
T Neuroblastoma 88 (Versteeg)
T Neuroblastic 64 (Delattre)
T Neuroblastoma 51 (Hiyama)
T Neuroblastoma 30 (Lastowska)
T Colon 290 (Sieber)
T Colon 315 (EXPO)
T Colon 38 (EXPO)
T Lung 114 (Bild)
T Lung 121 (EXPO)
T Breast 123 (Iglehart)
T Breast 351 (EXPO)
T Prostate 72 (EXPO)

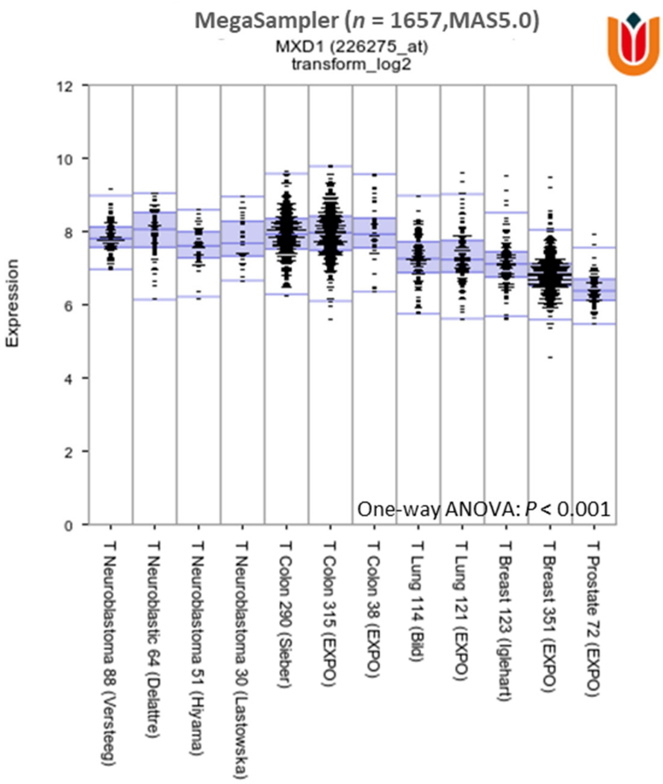
MegaSampler (n = 1657,MAS5.0)
MXD1 (226275_at)
transform_log2
Expression
One-way ANOVA: P < 0.001
T Neuroblastoma 88 (Versteeg)
T Neuroblastic 64 (Delattre)
T Neuroblastoma 51 (Hiyama)
T Neuroblastoma 30 (Lastowska)
T Colon 290 (Sieber)
T Colon 315 (EXPO)
T Colon 38 (EXPO)
T Lung 114 (Bild)
T Lung 121 (EXPO)
T Breast 123 (Iglehart)
T Breast 351 (EXPO)
T Prostate 72 (EXPO)

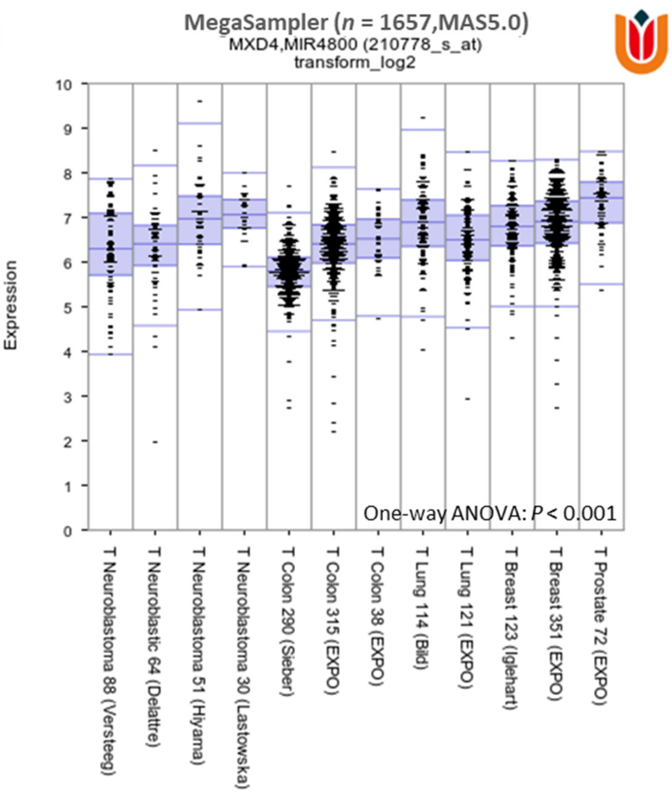
MegaSampler (n = 1657,MAS5.0)
MXD4,MIR4800 (210778_s_at)
transform_log2
Expression
One-way ANOVA: P < 0.001
T Neuroblastoma 88 (Versteeg)
T Neuroblastic 64 (Delattre)
T Neuroblastoma 51 (Hiyama)
T Neuroblastoma 30 (Lastowska)
T Colon 290 (Sieber)
T Colon 315 (EXPO)
T Colon 38 (EXPO)
T Lung 114 (Bild)
T Lung 121 (EXPO)
T Breast 123 (Iglehart)
T Breast 351 (EXPO)
T Prostate 72 (EXPO)

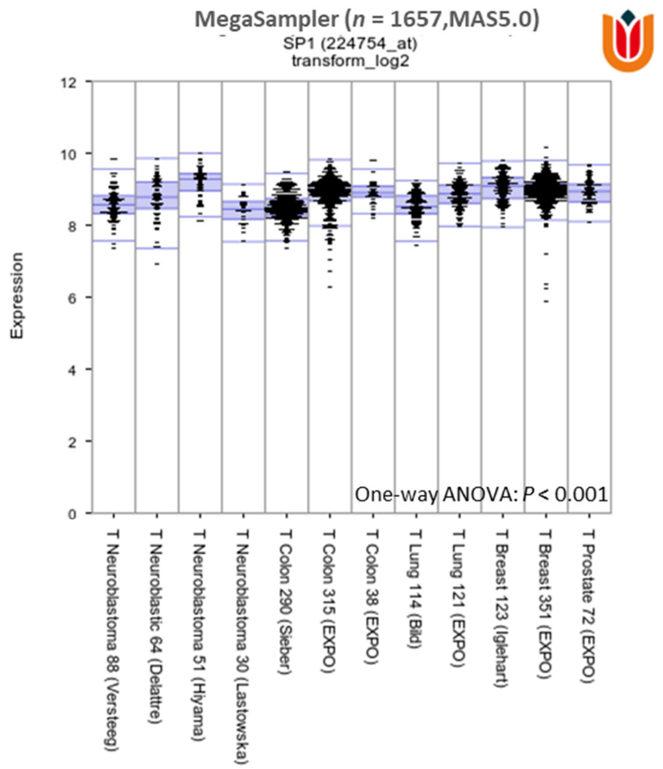
MegaSampler (n = 1657,MAS5.0)
SP1 (224754_at)
transform_log2
Expression
One-way ANOVA: P < 0.001
T Neuroblastoma 88 (Versteeg)
T Neuroblastic 64 (Delattre)
T Neuroblastoma 51 (Hiyama)
T Neuroblastoma 30 (Lastowska)
T Colon 290 (Sieber)
T Colon 315 (EXPO)
T Colon 38 (EXPO)
T Lung 114 (Bild)
T Lung 121 (EXPO)
T Breast 123 (Iglehart)
T Breast 351 (EXPO)
T Prostate 72 (EXPO)

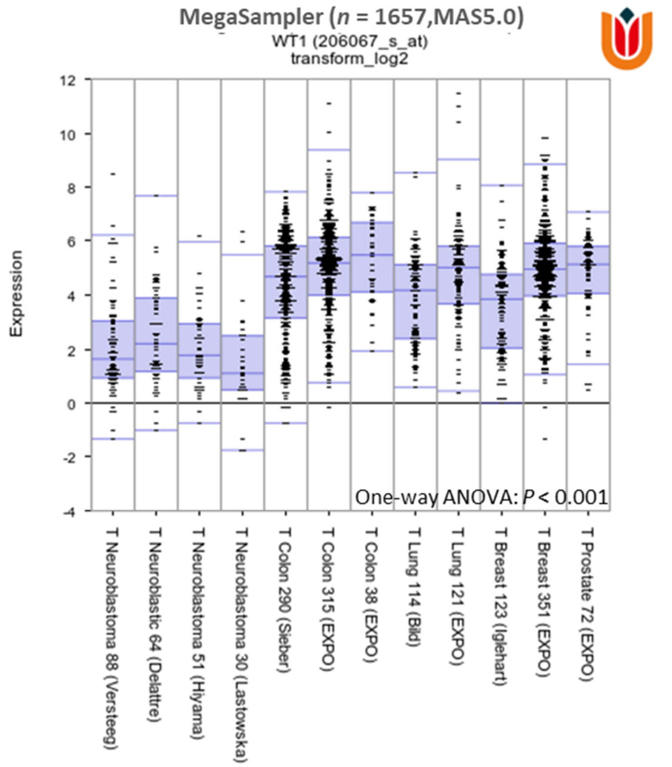
MegaSampler (n = 1657,MAS5.0)
WT1 (206067_s_at)
transform_log2
Expression
One-way ANOVA: P < 0.001
T Neuroblastoma 88 (Versteeg)
T Neuroblastic 64 (Delattre)
T Neuroblastoma 51 (Hiyama)
T Neuroblastoma 30 (Lastowska)
T Colon 290 (Sieber)
T Colon 315 (EXPO)
T Colon 38 (EXPO)
T Lung 114 (Bild)
T Lung 121 (EXPO)
T Breast 123 (Iglehart)
T Breast 351 (EXPO)
T Prostate 72 (EXPO)

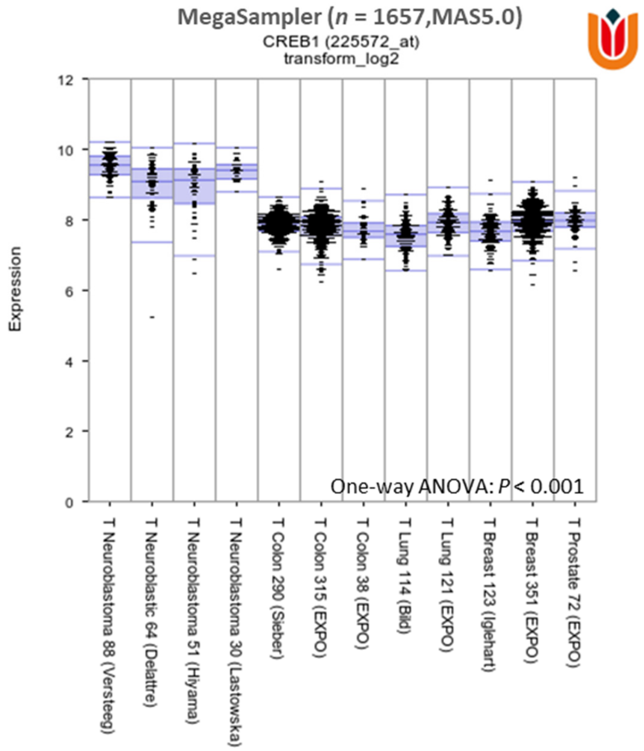

(B)

Tukey's post-hoc tests for multiple comparisons:

		ODC1	MYCN	MYC	MAX	MXD1	MXD4	SP1	WT1	CREB1
Neuroblastoma (Versteeg)	Breast (Iglehart)	<0.001	<0.001	<0.001	1.000	<0.001	<0.001	<0.001	<0.001	<0.001
	Breast (EXPO)	1.000	<0.001	<0.001	0.548	0.027	<0.001	0.919	0.749	<0.001
	Colon (Sieber)	1.000	<0.001	<0.001	0.548	0.027	<0.001	0.919	0.749	<0.001
	Colon (EXPO)	1.000	<0.001	<0.001	0.016	0.472	0.998	<0.001	<0.001	<0.001
	Colon Rectum (EXPO)	1.000	<0.001	<0.001	0.471	0.622	1.000	0.003	<0.001	<0.001
	Lung (Bild)	0.015	<0.001	<0.001	0.074	0.074	0.002	1.000	<0.001	<0.001
	Lung (EXPO)	0.024	<0.001	<0.001	0.572	<0.001	0.995	<0.001	0.007	<0.001
	Prostate (EXPO)	0.001	<0.001	<0.001	1.000	<0.001	<0.001	<0.001	<0.001	<0.001
Neuroblastoma (Delattre)	Breast (Iglehart)	<0.001	<0.001	<0.001	<0.001	<0.001	0.336	<0.001	<0.001	<0.001
	Breast (EXPO)	1.000	<0.001	<0.001	<0.001	0.809	<0.001	0.004	1.000	<0.001
	Colon (Sieber)	1.000	<0.001	<0.001	<0.001	0.809	<0.001	0.004	1.000	<0.001
	Colon (EXPO)	1.000	<0.001	<0.001	<0.001	1.000	0.002	0.803	<0.001	<0.001
	Colon Rectum (EXPO)	1.000	<0.001	<0.001	<0.001	0.992	0.728	0.613	<0.001	<0.001
	Lung (Bild)	0.160	<0.001	<0.001	<0.001	0.005	0.999	0.120	<0.001	<0.001
	Lung (EXPO)	0.222	<0.001	<0.001	<0.001	<0.001	0.706	0.844	0.653	<0.001
	Prostate (EXPO)	<0.001	<0.001	<0.001	<0.001	<0.001	0.361	0.148	<0.001	<0.001
Neuroblastoma (Hiyama)	Breast (Iglehart)	<0.001	<0.001	<0.001	0.746	0.015	1.000	0.862	<0.001	<0.001
	Breast (EXPO)	0.540	<0.001	<0.001	0.016	<0.001	<0.001	<0.001	0.777	<0.001
	Colon (Sieber)	0.540	<0.001	<0.001	0.016	<0.001	<0.001	0.000	0.777	<0.001
	Colon (EXPO)	0.717	<0.001	<0.001	<0.001	0.006	<0.001	0.000	<0.001	<0.001
	Colon Rectum (EXPO)	0.977	<0.001	<0.001	0.033	0.041	0.053	0.158	<0.001	<0.001
	Lung (Bild)	0.997	<0.001	1.000	0.998	1.000	0.999	<0.001	<0.001	<0.001
	Lung (EXPO)	0.999	<0.001	<0.001	0.024	0.303	0.016	<0.001	0.022	<0.001
	Prostate (EXPO)	<0.001	<0.001	<0.001	0.895	<0.001	1.000	0.109	<0.001	<0.001
Neuroblastoma (Lastowska)	Breast (Iglehart)	<0.001	<0.001	<0.001	0.005	0.005	1.000	<0.001	<0.001	<0.001
	Breast (EXPO)	0.005	<0.001	<0.001	<0.001	0.192	<0.001	0.999	0.035	<0.001
	Colon (Sieber)	0.005	<0.001	<0.001	<0.001	0.192	<0.001	0.999	0.035	<0.001
	Colon (EXPO)	0.002	<0.001	<0.001	<0.001	0.672	<0.001	<0.001	<0.001	<0.001
	Colon Rectum (EXPO)	0.045	<0.001	<0.001	<0.001	0.608	0.082	<0.001	<0.001	<0.001
	Lung (Bild)	<0.001	<0.001	0.137	0.927	0.896	0.996	0.984	<0.001	<0.001
	Lung (EXPO)	<0.001	<0.001	<0.001	<0.001	0.093	0.052	<0.001	<0.001	<0.001
	Prostate (EXPO)	1.000	<0.001	<0.001	0.017	<0.001	1.000	<0.001	<0.001	<0.001

Figure S9. Expression of the transcriptional regulators of ODC1, as identified via the DoRothEA interactions dataset available in OmniPath, across multiple tumour types. (**A**) The transcription factors (TFs) MYC, MXD1, MXD4, CREB1, SP1 and WT1 were identified as regulators of ODC1. Expression of these TFs, as well as ODC1 and MYCN, in neuroblastoma, colon, lung, breast and prostate cancer cohorts was analysed using the MegaSampler module in the R2 Genomics Analysis and Visualization Platform. The datasets are made up of samples from primary tumours, except for the EXPO datasets which may also contain non-primary tumours and tumours post-treatment. EXPO datasets are from the Expression Project for Oncology formed by the International Genomics Consortium. All datasets included in the analysis used the same chip type (u133p2), and were normalised by MAS5.0 to allow for comparisons. Accession numbers for the different datasets are as follows: T Neuroblastoma 88 (Versteeg), GSE16476; T Neuroblastoma 64 (Delattre), GSE12460; T Neuroblastoma 51 (Hiyama), GSE16237); T Neuroblastoma 30 (Lastowska), GSE13136; T Colon 290 (Sieber), GSE14333; T Colon 315 (EXPO), GSE2109; T Colon Rectum 38 (EXPO), GSE2109; T Lung 114 (Bild), GSE3141; T Lung 121 (EXPO), GSE2109; T Breast 123 (Iglehart), GSE5460; T Breast 351 (EXPO), GSE2109; T Prostate 72 (EXPO), GSE2109). For each TF analysed, a one-way ANOVA identified significant differences across the datasets. (**B**) Tukey's post-hoc tests for multiple pairwise comparisons were performed, and the significance between each adult cancer dataset and each neuroblastoma dataset are summarised. Significant differences are highlighted in grey.

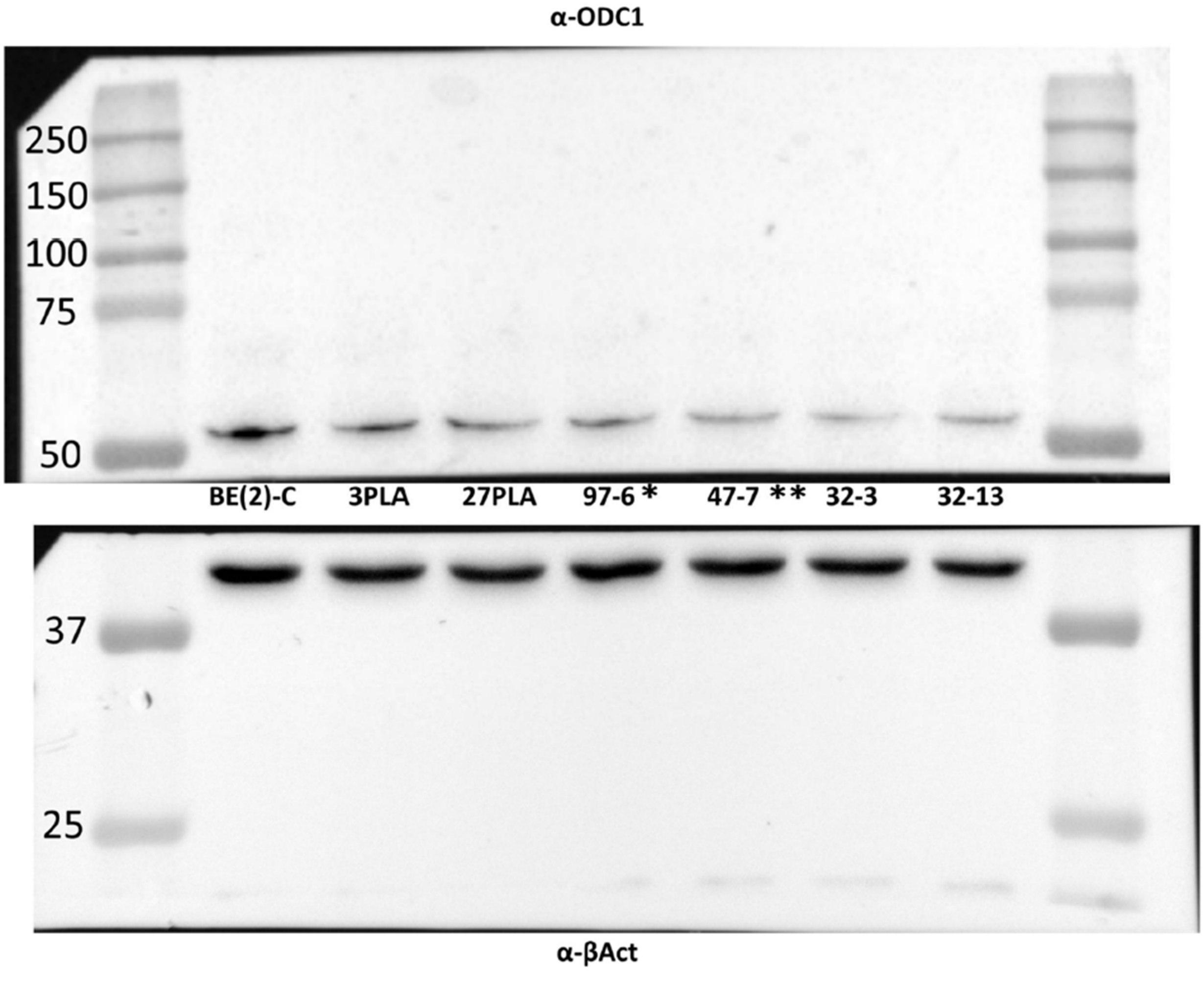

Figure S10. Uncropped WB of Figure 1C. Clones 97–6 and 47–7 have been used in this work as clones AG-1 (97–6) * and AG-2. (47–7) ** respectively.
